# Biomarkers and novel therapeutic approaches for diffuse large B-cell lymphoma in the era of precision medicine

**DOI:** 10.18632/oncotarget.27785

**Published:** 2020-11-03

**Authors:** Niraj Lodhi, Moe Tun, Poonam Nagpal, Arati A. Inamdar, Nehad M. Ayoub, Noor Siyam, Lucia Oton-Gonzalez, Angela Gerona, Dainelle Morris, Rana Sandhu, Kwangsun Stephen Suh

**Affiliations:** ^1^Department of Immunotherapeutic and Biotechnology, Texas Tech Health Science Center, Abilene, TX, USA; ^2^Formerly: The Genomics and Biomarkers Program, John Theurer Cancer Center at Hackensack University Medical Center, David Jurist Research Building, Hackensack, NJ, USA; ^3^College of Natural, Applied, and Health Sciences, Kean University, Union, NJ, USA; ^4^Department of Clinical Pharmacy, Jordan University of Science and Technology, Irbid, Jordan; ^5^Department of Medical Sciences, University of Ferrara, Ferrara, Italy; ^6^DiagnoCine, Hackensack, NJ, USA; ^*^These authors contributed equally to this work

**Keywords:** DLBCL, diagnosis, R-CHOP, precision medicine, prognosis

## Abstract

Despite the great efforts for better treatment options for diffuse large B-cell lymphoma (DLBCL) (most common form of non-Hodgkin lymphoma, NHL) to treat and prevent relapse, it continues to be a challenge. Here, we present an overview of DLBCL and address the diagnostic assays and molecular techniques used in its diagnosis, role of biomarkers in detection, treatment of early and advanced stage DLBCL, and novel drug regimens. We discuss the significant biomarkers that have emerged as essential tools for stratifying patients according to risk factors and for providing insights into the use of more targeted and individualized therapeutics. We discuss techniques such as gene expression studies, including next-generation sequencing, which have enabled a more understanding of the complex pathogenesis of DLBCL and have helped determine molecular targets for novel therapeutic agents. We examine current treatment approaches, outline the findings of completed clinical trials, and provide updates for ongoing clinical trials. We highlight clinical trials relevant to the significant fraction of DLBCL patients who present with complex cases marked by high relapse rates. Supported by an increased understanding of targetable pathways in DLBCL, clinical trials involving specialized combination therapies are bringing us within reach the promise of an effective cure to DLBCL using precision medicine. Optimization of therapy remains a crucial objective, with the end goal being a balance between high survival rates through targeted and personalized treatment while reducing adverse effects in DLBCL patients of all subsets.

## INTRODUCTION

Diffuse Large B-cell lymphoma (DLBCL), the most common subtype of lymphoma, accounts for up to 40% of non-Hodgkin adult lymphoma cases globally [1, 2]. DLBCL has a higher incidence among males, being the mean age at diagnosis approximately 70 years. However, it may occur in young adults and children as well [3, 4]. In the United States, the annual incidence of DLBCL is 7–8 per 100,000 people [3]. In Europe, the crude incidence is 3.8 per 100,000 people per year and is around 10.2 per 100,000 adults in the UK [3, 5]. The incidence of DLBCL usually increases with age. DLBCL has reported many risk factors, such as: family history of hematologic malignancy, autoimmune disease, HIV and hepatitis C virus infection, a high body index during young adulthood, and occupational and/or environmental exposure to certain drugs or chemicals [5, 6].

DLBCL is an aggressive lymphoma marked by significant heterogeneity of clinicopathologic and molecular genetic features. It is characterized by a diffuse proliferation of large and mature B-cells, typically larger than average macrophages and lymphocytes, sometimes twice as large [7]. DLBCL can arise *de novo* or transform from an already existent, less aggressive lymphoma, such as follicular lymphoma or small lymphocytic lymphoma [8, 9]. Based on the anatomic site of occurrence, DLBCL is classified into different subtypes, including Primary Central nervous system lymphoma (PCNSL) DLBCL, primary cutaneous DLBCL, leg type, and intravascular large B-cell lymphoma [10].

PCNSL accounts for approximately 2% of all primary central nervous system tumors. PCNSL is a uncommon, but aggressive type of non-Hodgkin extranodal lymphoma (NHL). It is limited to the eyes, brain, spinal cord or leptomeninges [11, 12]. The 5- and 10-year survival rates for PCNSL are 29.9% and 22.2%, respectively [11]. DLBCL constitutes 90% of all PCNSL cases, the remaining percentage belonging to T-cell, Burkitt’s, lymphoblastic and low-grade lymphomas [11, 13]. Common extranodal sites (primary extranodal lymphomas) include bone, breast, thyroid, CNS, testicles, and Primary Vitreoretinal Lymphoma (PVRL) [7]. While 10–15% of primary DLBCL arises in several sites, the lower leg, on one or both, remains the main part of insurgence. Usually patients present red or bluish-red tumors and from there it disseminates to other sites [14].

Different morphological variants of DLBCL include: EBV-positive DLBCL or Not Otherwise Specified (NOS), T-cell/histiocyte rich large B-cell lymphoma, Primary Mediastinal (thymic) Large B-cell Lymphoma (PMLBL), plasmablastic lymphoma and primary effusion lymphoma [15–17]. Elderly EBV-positive DLBCL occurs in patients over 50 years of age with a prior lymphoma history or immunodeficiency [18]. Among these patients, 70% have extranodal involvement, most commonly skin, lung, tonsil, and stomach with or without lymph node (LN) involvement. The remaining 30% present with LN involvement only. A significant proportion of DLBCL cases remain biologically heterogeneous and do not fit into any specific disease sub-group; these are defined as Diffuse Large B-cell Lymphoma-NOS (DLBCL-NOS) [7].

DLBCL can be subdivided into several types on the basis of cytological and molecular features. Anaplastic, centroblastic and immunoblastic are the three common morphological variants of DLBCL [8]. In general, centroblastic lymphoma has improved prognosis than immunoblastic or anaplastic types [8]. As improvements have accrued in technologies such as gene expression profiling (GEP), the biology of DLBCL-NOS has become better understood, providing new insights and leading to the identification of two principal molecularly distinct groups: germinal center B-cell-like (GCB-DLBCL) and non-GCB-like, of which most of the latter have a B-cell-like phenotype (ABC-DLBCL) which is activated [13]. The non-GCB group has a more aggressive clinical course than GCB, and is associated with substantially worse outcomes when treated with R-CHOP (rituximab, cyclophosphamide, vincristine, doxorubicin, and prednisone).

GCB-DLBCLs are heterogeneous and are characterized by expression in B-cell lymphoma 6 (BCL-6), a transcriptional repressor, and/or overexpression of B-cell lymphoma 2 (BCL-2), an anti-apoptotic protein, are commonly seen in GCB- DLBCLs [15, 19, 20]. ABC-DLBCLs have a gene signature similar to activated peripheral blood B-cells. In addition to mutations in BCL-6 and BCL-2, approximately 30–40% of GCB-DLBCLs have t(14;18) translocation, 30% have c-rel amplification, 20% have mutations of EZH2, and 10% have a deletion of PTEN [13]. None of these mutations are seen in ABC-DLBCL, except for BCL-2 overexpression, although overexpression of BCL-2 ABC-DLBCL occurs *via* a different mechanism(s) [15].

**Figure 1:**
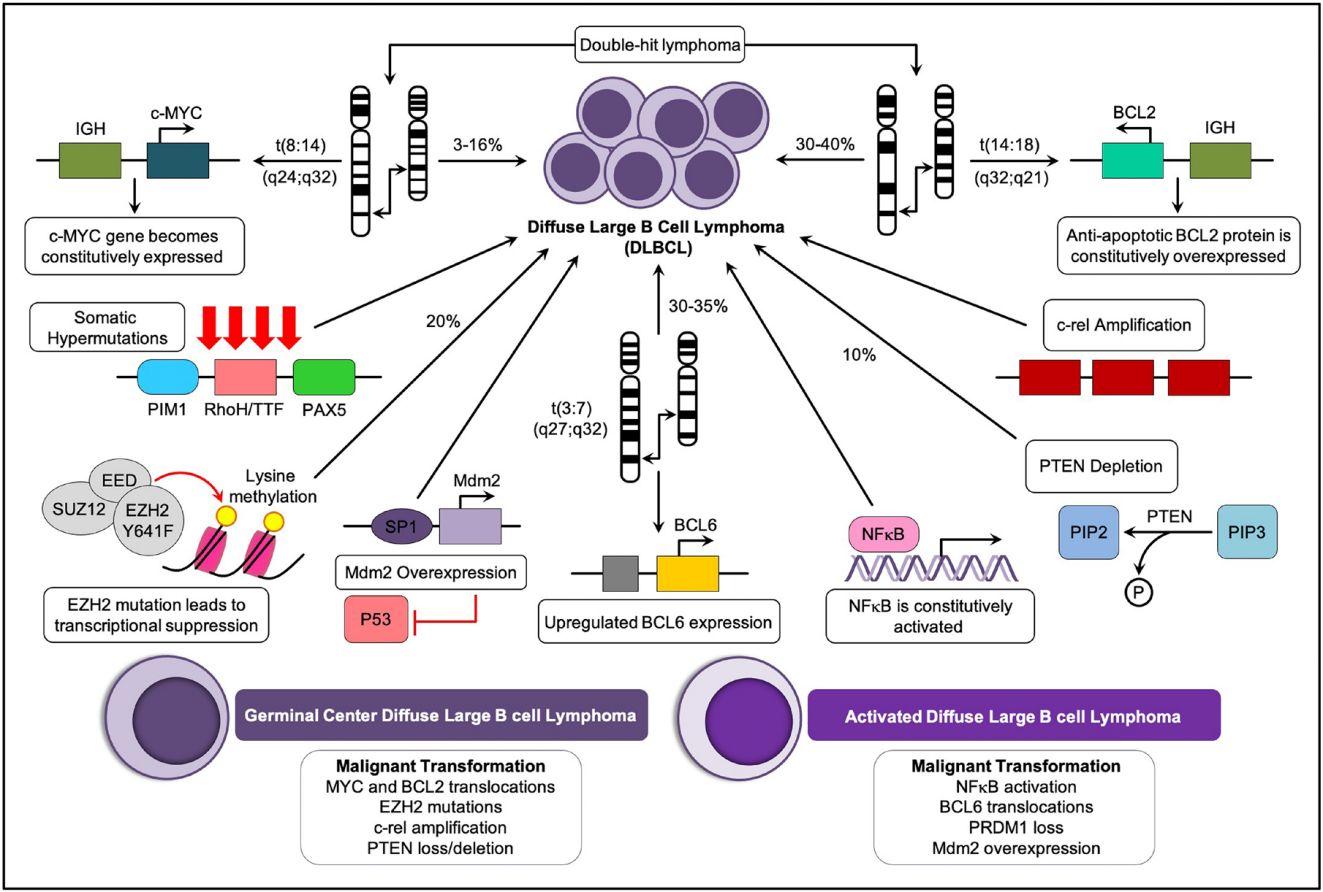
Key oncogenetic pathways and major molecular subtypes of DLBCL. DLBCL may arise from multiple oncogenetic alterations in B-cells. The main oncogenic pathways involve the translocation of genetic material, gene amplification, and somatic hypermutations (SMH). The two major molecular subtypes of DLBCL, the germinal center, and the activated type, are listed.

The incidence of ABC DLBCL is higher in older patients and represents about 40% of all DLBCL cases [21]. The main identifying feature of ABC-DLBCLs is a constitutive expression of the NF-κB signaling pathway due to aberrations in the components of the CBM signaling complex, which consists of caspase recruitment domain 11 (CARD11), BCL10, and MALT1, and which promotes proliferation, cell survival, and inhibition of tumor cell apoptosis [22]. The pathological and immunological details of DLBCL are described in Figure 1. Other two subtypes represented by Double-Hit Lymphomas (DHL) (5–10% of all DLBCL lymphomas) and Double-Expressor Lymphomas (DEL), present MYC and BCL2 protein overexpression, together with the GCB and ABC subtypes [23]. DHL and DEL are associated to poor prognosis and in particular, DHL usually presents chromosomal rearrangements [24]. Both subsets overlap with the ABC and GCB molecular subtypes. DEL is more common among ABC, and the actual driver of ABC's worse prognosis [25], while DHL is more common in GCB. DHL patients present an overall survival of 12 months or less and usually respond poorly to R-CHOP standard therapy [26, 27]. Notably, most of these lymphomas belong to the GCB subgroup, which is predicted to have a better prognosis with R-CHOP treatment.

Diagnosis of DLBCL is made from the examination of blood, bone marrow, and affected lymph nodes and organs, and is usually based on the typical morphology of the DLBCL phenotype as reported by lymph node biopsy, immunohistochemistry (IHC), flow cytometry, and cytogenetic studies. Flow cytometry can be used to detect light chain restriction and to diagnose expanded populations of B-cells, which is useful since flow cytometry can be used even when IHC fails to detect BCL-2 overexpression or t(14;18) translocation [28].

The differential diagnosis of DLBCL includes discrimination from nonmalignant conditions such as infectious mononucleosis, non-lymphoid malignancies, and other lymphoma subtypes, especially NHL, lymphoblastic lymphoma, and Burkitt lymphoma [27]. The presence of typical DLBCL features based on molecular and/or immunophenotypic studies aid in diagnosis in most cases (Figure 1).

R-CHOP (Rituximab- Cyclophosphamide, doxorubicin, vincristine, and prednisone) is considered the gold standard treatment for DLBCL and is commonly used [21]. Clinical investigators have tried continuously to improve its effectiveness by adding novel drugs and proposing new combinations. Although treatment regimens with or without R-CHOP have been assessed in various clinical trials, the R-CHOP regimen administered every 21 days has emerged as the standard-of-care for DLBCL. Research on newer and novel therapeutic agents targeting precise disease component(s) based on advances in our understanding of the relevant oncogenic pathways is opening the door for more effective treatments for DLBCL, which promise to achieve better survival rates and lower recurrence rates. Improvements in prognostic accuracy based on the availability of predictive biomarkers will be crucial for the development of individualized risk-adapted therapy.

## UTILIZATION AND COST OF HEALTHCARE RELATED TO DLBCL

The Cost-effectiveness of CHOP and R-CHOP (CHOP plus rituximab) has been extensively compared, but data from clinical trials is difficult to extrapolate to general DLBCL patients since these trials only include patients in curative intent. To bypass this, an administrative approach has been used in Ontario (Canada) [29]. The interpretation of the results of this population-based retrospective cohort study was that addition of rituximab to standard CHOP chemotherapy supposed an increase in survival rates at higher costs, and for patients under 60 years old it was potentially cost-effective under standard thresholds. Nevertheless, cost-effectiveness decreased significantly with age. Data from this study suggested that rituximab, for the treatment of elderly patients, may not be of economic interest [30]. This conclusion has important clinical implications regarding age-related use and funding decisions on this drug [30].

Another finding from a comparison of Medicare, in the United States, was that patients that relapsed had higher rates and costs for the use of healthcare compared to those that did not relapse. [31]. The research, presented at the 58th Annual Meeting & Exposition of the American Society of Hematology, suggests that improvements in first-line DLBCL therapy can offer “important savings in healthcare together with improved clinical outcomes.” [31]. In this regard, total healthcare costs related to the relapsed cohort were $4848 per patient per month compared to $1427 per patient per month among non-relapsed patients, more than three times higher. The major cost drivers of this disparity among relapsed and non-relapsed patients were total outpatient care ($2984 vs. $632) and inpatient costs ($1220 vs. $443) [31]. Among relapsed patients, the total costs from the date of relapse to the end of the study were double the costs during remission [31]. Chimeric antigen receptor T cell immunotherapy (CAR-T therapy) (discussed below under the section TREATMENT OF RELAPSED/REFRACTORY DLBCL) is emerging immunotherapy for different pediatric and adult leukemias and lymphomas. Potential T-cell treatments will have an average cost ranging from $300,000 to $500,000 per patient [32, 33].

## MOLECULAR TECHNIQUES IN DLBCL DIAGNOSIS

DLBCL diagnosis involves a combination of clinical, laboratory, pathological, and radiological assessments. Detecting the disease early will require a multi-targeted approach that integrates diagnostic biomarkers, cytogenetic studies, and other novel diagnostic assays. Diagnostic and prognostic biomarkers in DLBCL (Supplementary Table 1) have emerged as important tools for stratifying patients according to risk factors and for creating personalized treatment regimens. Immunophenotyping using Immunohistochemistry (IHC) and flow cytometry and cytogenetic analysis using fluorescent *in situ* hybridization (FISH) and PCR are commonly used to diagnose DLBCL [34]. Other techniques, such as next-generation sequencing (NGS), gene expression profiling (GEP) or high-resolution array comparative genomic hybridization (array CGH) are also available but are not part of routine clinical practice [35, 36]. These advanced techniques are applicable only for the identification of the mutation in genes or aberration in gene expression for research or diagnosis purposes; array CGH and NGS, respectively, but are not widely used in clinical studies. Streamlined integration of these various methodologies in routine clinical practice is an essential step towards improving DLBCL diagnosis and subsequent outcomes [37]. Supplementary Table 1 lists the various diagnostic assays and antibodies that are currently available for the detection of DLBCL and its biomarkers. However, many commercially available tests are just intended for research use. More information about these products and how they are interpreted for diagnostic purposes is presented in depth in Supplementary Table 1.

The National Comprehensive Cancer Network (NCCN) guidelines for diagnostic workup of DLBCL patients include: (a) pathological review of lymph node biopsy slides for morphological variants for DLBCL (i.e., centroblastic, immunoblastic, and anaplastic); (b) IHC for CD20, CD3, CD5, CD10, CD45, BCL-2, BCL-6, Ki-67, IRF4/MUM1, and MYC; (c) flow cytometry for kappa/lambda, CD45, CD3, CD5, CD19, CD10, and CD20; (d) PCR for IgH and TCR gene rearrangements, and (e) FISH for major translocations, such as t(8;14), t(14;18), and t(3; v) [30, 38]. Differentiating between subtypes is especially important for maximizing clinical outcomes since it allows for a more targeted and precise treatment regimen. Different subtypes can be identified *via* an additional IHC panel consisting of Cyclin D1, kappa/lambda, CD30, CD138, EBER-ISH, ALK, and HHV8 (primary effusion lymphoma). Certain circumstances may require cytogenetics or FISH for analysis of t(14; 18), t(3; var), t(8; 14), and t(8; var) [38].

The Cell of Origin (COO) profiling is based on the analysis of gene expression to identify patterns characteristic of particular populations of cells that bear important medical, prognostic and biological consequences. Expression patterns typical of germinal core B-cells (GCB group), activated B-cells (ABC group), and other non-classified subtypes, for example [39–41]. Various algorithms have been developed based on the protein expression patterns as detected by IHC. For reference, the Hans algorithm uses the expression of CD10, BCL-6 and MUM1 to classify DLBCL into GCB or non-GCB subtypes [41–43].

Given that DLBCL is the most prevalent subtype of non-Hodgkin lymphomas, there is a need for the integration of more biomarkers specific to DLBCL into currently available lymphoma panels. Furthermore, additional development of clinical applicability of currently available research-use-only panels is necessary so they can become available for clinical use. The HTG EdgeSeq Lymphoma Panel for example tests the expression of 92 genes commonly examined in lymphoma. Of these, 22 are common NHL B-cell lymphoma markers. Although this panel effectively consolidates biomarker measurement, it could be improved by integrating more DLBCL-specific biomarkers. The inclusion of major DLBCL biomarkers such as MYC, NF-κB, CD43, MDM2, and Ki-67 could provide a more thorough and effective diagnosis of DLBCL.

Such chromosome analysis is an essential component of diagnosing and determining the stages of lymphomas. Through cytogenetic studies, DLBCL patients have been found to exhibit a wide variety of chromosomal irregularities. A 2013 study examined the relationship between frequent chromosomal abnormalities and prognosis in patients with DLBCL [44]. Using chromosome banding, the study demonstrated that of eleven different chromosomal abnormalities, just a 17p loss (loss of p53) showed a substantial correlation with a poorer long-term prognosis. The overall survival (OS) and progression-free survival (PFS) frequencies for patients with a 17p loss were 32% and 27%, respectively. Patients without loss of 17p (i.e., possessing wild-type p53) exhibited frequencies of 67% and 59%, respectively [37, 44].

Although G-banding has proven to be useful for detecting specific rearrangements that provide insight into clinical outcomes, this technique has been problematic for identifying more complex abnormalities. A newer cytogenetic technique, spectral karyotyping (SKY), offers a more precise and thorough picture of chromosomal irregularities. SKY has enabled the detection of breakpoints in DLBCL that were not previously identifiable *via* G-banding. Of the new recurring breakpoints identified *via* SKY, breaks at 16q11-13, 12p11, and 11p11 were the most common. In addition, four new translocations were identified: der (14) t(3; 14) (q21; q32), t(1; 13) (p32; q14), t(1; 7)(q21; q22), and der (6) t(6;8) (q11; q11) [45]. Integrating interphase FISH (iFISH) with SKY has been recommended for identifying translocations and diagnosing DLBCL [46].

Tissue microarray-based FISH has been utilized in DLBCL to assess the role of BCL-6 rearrangement on the outcome for patients treated with CHOP or R-CHOP [47]. Expression of other genes, including PATZ1, MYC-3, and RUNX3, as well as microRNAs and non-coding RNAs, have also been assessed for their prognostic and diagnostic utility in DLBCL [48–51]. Comparative arrays of genomic hybridisation and single nucleotide polymorphism (SNP) arrays are often used to distinguish between DLBCL subtypes [52]. Array CGH highlights patterns of deletions or amplification and can determine allelic ratios or loss of heterozygosity, although it cannot detect balanced translocations [52]. The GCB-DLBCL subtype is associated with REL locus amplification, BCL-2 translocation and Ig loci Somatic Hypermutation (SHM) amplification [52]. In fact, SHM is recognized in both of GCB and ABC types, and the pattern is different, which constitutes a marker for DLBCL molecular classification and delineates the Cell Of Origin (COO) in DLBCL. In a study Alkodsi et al. (2019), expression of thirty-six SHM target genes stratified DLBCL into four novel SHM subtypes, and each subtype having a distinct clinical outcome within each of the COO subtypes [53].

The quantitative Nuclease Protection Assay (qNPA) and nanoString nCounter platform have proved robust technologies in recent years that can quantitatively calculate gene expression from FFPE specimens [54, 55]. In the qNPA, targeted probes are hybridized to mRNA released from FFPE samples. Non-hybridized mRNA is removed by nuclease digestion, and the mRNA/probe duplexes are secured to a plate by oligonucleotide linkers. The mRNA is then linked to a chemiluminescent substrate, which is imaged to measure gene expression levels. In the NanoString nCounter assay, probes are used to capture and count the number of targeted genes.

The nanoString platform can also be used to identify gene translocations with 5′ and 3′ probes. These technologies are advantageous because they require minimal RNA input, and use probe-to-target hybridization, which eliminates the need for snap-frozen materials, cDNA synthesis, or amplification. The qNPA technology relies on the acquisition of gene expression based on chemistry, and nanoString nCounter is a platform for digital expression. They have both been shown to be effective and accurate for assigning cell of origin from FFPE tissue. NanoString nCounter has an advantage over qNPA because it can profile a significantly larger number of genes [41, 55]. But these techniques are yet to be adapted in regular clinical practice.

Together, these advanced and uniquely designed genetic and molecular techniques have aided in the identification of underlying molecular mechanisms contributing to the pathogenesis and clinical progression of DLBCL. They have also contributed to our ability to predict clinical outcomes of lymphoma patients, and have been shown, as well, to identify targets for novel therapeutic agents.

## ROLE OF BIOMARKERS IN DETECTION AND DIAGNOSIS

Identification of disease-based biomarkers has become a central component of research supporting the diagnosis and prognosis of almost all types of human diseases, including DLBCL. Biomarkers are essential elements of underpinning guidelines for assessing risk, screening, prognostic determination, treatment response, and monitoring disease progression. The identification of several biomarkers for DLBCL through IHC, FISH, flow cytometry, western blot, NGS (including whole-genome/whole-exome sequencing), enzyme-linked immunosorbent assay (ELISA), and polymerase chain reaction (Supplementary Table 2) has provided remarkable insight into disease pathogenesis and mechanisms. The details of some of the critical biomarkers are discussed in the following subsections.

### B-cell lymphoma 2 (BCL-2)

**Figure 2:**
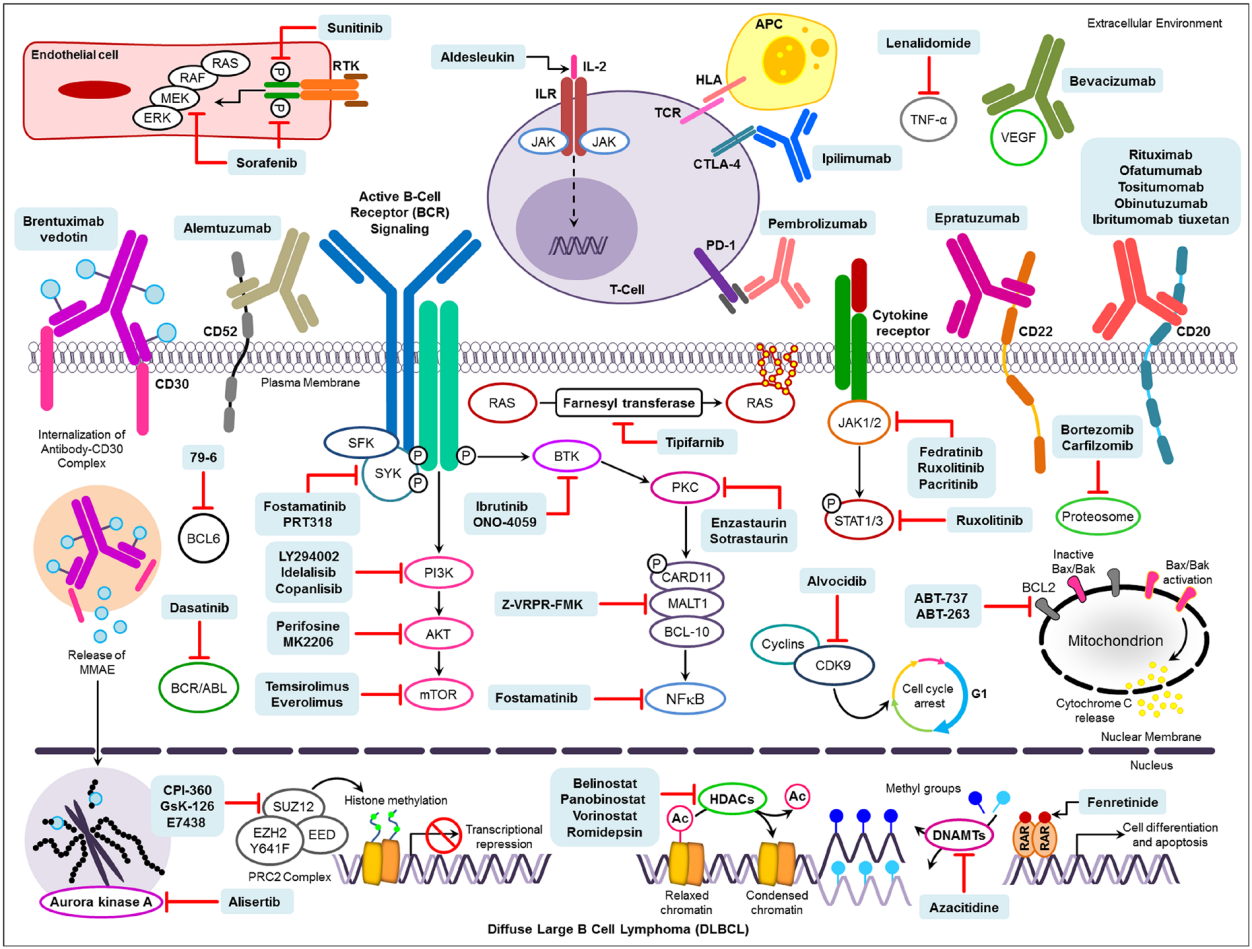
Novel drug targets and pharmacological therapies under investigation for the treatment of DLBCL. Abbreviations: RTK: Receptor tyrosine kinases; IL-2: Interleukin 2; ILR: Interleukin receptor; TCR: T-cell receptor; HLA: Human leukocyte antigen; APC: Antigen-presenting cells; TNF-α: Tumor necrosis factor-α; VEGF: Vascular endothelial growth factor; Ac: Acetyl group. HDACs: Histone deacetylase; DNAMTs: DNA methyltransferases; RAR: Retinoic acid receptor.

BCL-2, an oncogenic biomarker, and among the first known members of the BCL-2 family of regulator proteins located on the mitochondrial outer membrane (Figure 2) [5]. BCL-2 promotes cell survival *via* inhibition of apoptosis (Supplementary Table 2). The BCL-2 family, Bax, and Bak pro-apoptotic proteins allow cytochrome C and ROS to be released through membrane permeability and later act as signals during the apoptotic cascade and are inhibited by BCL-2 itself [56]. DLBCL samples often exhibit the BCL-2 chromosomal translocation t(14;18), detected by FISH, which results in upregulation of BCL-2 expression, resulting in BCL-2-mediated resistance to apoptotic stimuli [5, 57].

Two mechanisms suppress apoptosis by other family members of BCL-2 myeloid cell leukemia 1 (MCL-1), extra-large BCL (BCL-xL), and BFL-1; Second, these proteins attach and prevent the oligomerisation of the proapoptotic effector proteins, BAX and BAK, which trigger apoptosis by forming pores in the outer membrane of the mitochondria releasing cytochrome c [58–60]. Additionally, the antiapoptotic BCL-2 family members bind and sequester the proapoptotic BH3-only activator proteins, BCL2 interacting mediator of cell death (BIM) and BID, which activate BAX and BAK. Cancer cells adopt anti-apoptotic defense mechanisms in response to anti-cancer therapy. Cancer care may be enhanced by using anti-apoptotic inhibitors, and combining them with other anti-cancer agents.

The cells are immortalised due to the increased expression of BCL-2. Enhanced expression of BCL-2 in DLBCL is associated with poor prognosis and shorter survival [61, 62]. BCL-2 t(14;18) is present in 20–30% of DLBCL cases (Figure 1) and is often observed in GCB-DLBCL-like variants [63]. Adding rituximab to standard chemotherapy overcomes BCL-2 ‘s influence on adverse prognosis [64, 65]. In cases of “double hit lymphoma” (DHL) where a BCL-2 translocation is accompanied with translocation of MYC, such as t(8;14) for MYC and t(14;18) for BCL-2, the prognosis is consistently poor [62]. Patients with lymphoma cells co-expressing MYC and BCL-2 have been shown to respond better to ABT-737 (a selective inhibitor of BCL-2, BCLxL, and BCLw) (Figure 2), showing that BCL-2 has a critical role in DHL [66, 67].

### B-cell lymphoma 6 (BCL-6)

BCL6 is among the most frequently rearranged gene, with 19% affecting all DLBCL patients [68]. It is a zinc-finger transcription factor that represses gene transcription in Germinal Center (GC) B-cells through the recruitment of co-repressor proteins and inhibits DNA damage response pathways and thus prevents arrest and apoptosis of cell cycles [69]. It is a regulator of processes involved with the clonal expansion of B-cells (Supplementary Table 2). In lymphoid malignancies, chromosomal translocations and mutations lead to BCL6 deregulation. Mice engineered to express constitutively BCL6 develop DLBCL similar to human disease in GC B cells [70, 71]. For most patients with active B cell lymphomas, BCL6 is often expressed constitutively due to translocation of heterologous promoter elements or promoter point mutations in the BCL6 locus [72, 73]. These changes include translocations fusing its coding sequence to heterologous promoters [74], point mutations in BCL6 promoting negative regulatory elements [72], or mutations that affect BCL6 transcription [50] acetylation-mediated BCL6 inactivation [75].

Aberrant blockage in the repressive function of BCL-6 is known to contribute to genetic instability, ultimately leading to neoplastic transformation [47, 76]. BCL-6 also auto-regulates its own transcriptional expression and indirectly up-regulates the expression of certain genes that are essential for GC reactions [77]. One of the genes directly targeted by BCL-6 is PR domain containing 1 lymphocyte-induced maturation protein 1 (BLIMP1) with zinc finger domain (PRDM1)/B [75], whose expression is required for terminal differentiation of GC B-cells to plasma cells [53]. PRDM1 is frequently inactivated specifically in ABC-DLBCL. The Pasqualucci et al. (2006) and Wagner et al. (2011) group findings indicate that BCL-6 deregulation and inactivation of PRDM1/BLIMP1 represent alternative pathogenetic pathways, all leading to a blockage of post-GC differentiation and eventually causing lymphomagenesis [75, 78, 79]. The translocation and hypermutation of BCL-6 at chromosome 3q27 with t(3;7) (q27;p12), translocations have been reported in 30–35% of DLBCL cases (Figure 1) and is a known event associated with the longer survival of malignant genetically unstable transformations associated with DLBCL [47]. This chromosomal region is also a frequent site for somatic mutations. Studies have indicated that BCL-6 rearrangement is associated with poorer outcomes in patients treated with R-CHOP [47]. In another study by [80], patients with poor prognosis had BCL-6 rearrangements together with BCL-2 deregulation and MYC translocation, further evidence that BCL-6 rearrangement is rarely found as a sole genetic abnormality in DLBCL.

### MYC

The MYC oncogene is a “master regulator” of cellular metabolism and proliferation. It is activated by a large number of oncogenic pathways, and in turn, stimulates many of the genetic changes that result in malignant transformation [81]. Translocations of MYC (Figure 1) were first associated with Burkitt lymphoma, but it has since been found that MYC recombination with other genes is reported in 3–16% of DLBCL cases [57, 60]. MYC, a transcription factor, is typically activated by WNT, Sonic hedgehog (Shh), EGF, and apoptotic signaling pathways. Importantly, it downregulates BCL-2 during apoptosis and regulates the expression of genes affiliated with cell proliferation. In GCB-DLBCL, the frequently observed t(8;14) (q24;q32) translocation involves rearrangement of the MYC gene, which impairs its normal regulation and results in its upregulation [57, 62, 63]. In addition, the fusion of MYC and Ig (heavy chain gene in t(8;14) translocations or light chain genes, kappa in t(2;8) translocations, or lambda in t(8;22) translocations) is also known to cause overexpression of MYC in DLBCL. In a meta-analysis study, the consequences of MYC translocations were not overcome by rituximab treatment [82]. The MYC translocation shows promise in patients with DLBCL treated with R-CHOP therapy as a prognostic factor although these results require confirmation [57]. The presence of a MYC rearrangement in patients is often correlated to bad outcomes [83, 84].

### Nuclear Factor kappa-B (NFκ-B)

NF-κB is a transcription factor that regulates the expression of the Ig kappa light chain and influences a wide range of biological processes including innate and adaptive immunity, inflammation, stress response, development of B-cells, and lymphoid organogenesis. [85]. It is well known that activation of the nuclear factor kappa-B (NF-κB) is essential for the growth and survival of tumor cells. Typically, lymphoid malignancies avoid cell death by constitutive activation of the NF-κB pathway which increases cell proliferation [86, 87].

Aberrant canonical NF-κB pathway activation in DLBCL attributes a significant fraction of the presence of oncogenic mutations in genes [88–90]; however, the previously published paper reveals that up to 15% of DLBCLs have genetic mutations activating the alternative NF-κB pathway. In this context, it should be noted that in addition to the ~10% DLBCL cases showing nuclear NF-κB activity solely for the alternative pathway (indicated by nuclear staining of p52 but not p50) and ~20% of DLBCLs showing nuclear staining for both p50 and p52 [88], this indicates activation of both canonical and alternative NF-κB pathways [91].

ABC DLBCLs engage the classical NF-κB pathways because they have rapid phosphorylation and turnover of IκBα and prominent nuclear accumulation of p50/p65 heterodimers with a lesser accumulation of p50/c-rel heterodimers [92]. RelA/p65 and p50 are the most common NF-κB subunits and involved in the classical NF-κB pathway. RelA / p65 nuclear over-expression in early stage DLBCL patients is correlated with significant poor survival [93]. Gene expression profiling analysis suggested immune dysregulation and anti-apoptosis may be relevant for the poorer prognosis associated with p65 hyperactivation in germinal center B-cell–like (GCB) DLBCL and also in activated B-cell–like (ABC) DLBCL, respectively [93]. In B-cells, numerous receptors, including the BCR, CD40, the B-cell-activating factor (BAFF) receptor, and various Toll-like receptors (TLRs) are are able to activate the NF-kB pathways. [86, 94, 95]. Alternatively, activation of NF-κB results from proteasome degradation of its inhibitor [inhibitor of kappa B (IκB)]. The activation of NF-κB is considered the hallmark of ABC-DLBCL [96]. More NF-κB target genes are expressed in ABC-DLBCL than in GCB-DLBCL; thus, ABC-DLBCL lines are more susceptible to inhibition of the NF-κB pathway. Increased NF-κB activity in ABC-DLBCL is stimulated by mutations in CARD11, a part of the CBM complex (CARD11, BCL-10, and MALT1), which acts as a signaling hub for activation of the classic NF-κB pathway downstream of the antigen receptors in B and T cells (Figure 2) [97, 98].

Clinically, it has been observed that ABC-DLBCL patients are less responsive to standard immuno-chemotherapy than other DLBCL subtypes, the NF-κB may be responsible for this, due to its ability of antagonizing the tumor suppressor activity of chemotherapeutic agents. Bortezomib, a proteasome inhibitor blocking degradation of IκBα (an inactivator of NF-κB), showed clinical benefits in relapsed ABC-DLBCL patients when combined with DA-EPOCH-R (Etoposide, Prednisone, Vincristine, Cyclophosphamide, Doxorubicin, Rituximab) [59, 64]. Moreover, pharmacological inhibitors of NF-κB activity, such as lenalidomide (Supplementary Table 5), have shown selective activity in non–GCB-DLBCL [59, 64].

### Mouse double minute 2 homolog (MDM2)

The negative regulator of the p53 tumor suppressor pathway; MDM2, regulates cell division and prevents tumor formation. In particular, MDM2 acts on its key target by repressing transcriptional activity *via* binding to the N-terminal trans-activation domain of p53 [79, 99]. The intracellular p53 levels are regulated by a self-regulatory feedback loop consisting of p53 and MDM2 [100, 101]. MDM2 has often been found to be over-expressed in cancer [102, 103]. In many tumors such as DLBCL, MDM2 overexpression is not solely caused by gene amplification, but is also indirectly associated with an increased affinity for the transcriptional activator SP1 (Figure 1), the binding of which results in elevated MDM2 expression in a gender-specific (females) and hormonal-dependent manner [100]. In DLBCL, MDM2 overexpression facilitates B-cell lymphomagenesis *in vivo* through the inactivation of wild type p53 tumor suppressor function [100, 101]. A single nucleotide polymorphism (T to G change) in MDM2 promoter region at position 309 (SNP309) affects MDM2 transcription [104]. Furthermore, an analysis of SNP309 in 108 patients treated with R-CHOP showed that, although the presence of the SNP did not correlate with poorer survival in DLBCL patients, p53 or MDM2 overexpression correlated with significantly worse survival when MUT-p53 was present [100, 101, 104].

### MicroRNA

MicroRNAs (miRNAs) are 17–25 nucleotide RNA molecules which regulate gene expression at the post-transcriptional level. The association of miRNA with mRNA leads to cleavage, suppression of stability, or translational repression of mRNA targets. Because miRNA target sites may be shared among groups of genes, a single miRNA can regulate the production of multiple proteins [105, 106]. Dysregulated miRNAs have been detected in several cancers of different histotypes [107]. Multiple miRNAs contribute to the pathogenesis of DLBCL. The down-regulation of miR-29 due to a translocation at t(3;7)(q27;q32) (Figure 1) involves the fusion of BCL-6 to a noncoding region of FRA7H, which suggests that miRNA replacement therapy might be effective for the treatment of DLBCL cases associated with loss of a specific miRNA [106, 108]. Other essential miRNAs in DLBCL are miR-155 and miR-17-92 which have DLBCL expression patterns that differentiate tumor cells from non-malignant B-cells [109]. Also, the expression of miR-155, miR-21, and miR-221 differs between ABC-DLBCL and GCB-DLBCL subtypes (see Supplementary Table 2), indicating the specificity of miRNA expression in DLBCL [83]. MiR-21 expression in tumor cells and serum is negatively associated with DLBCL patient prognosis since the knockdown of miR-21 by the use of antisense oligonucleotides significantly increased the cytotoxic effects of the CHOP treatment regimen in human peripheral DLBCL blood cells [105, 108]. MiRNA profiling has shown that a seed sequence mutation of miR-142 (found in approximately 20% of DLBCL patients) plays a role in pathogenesis, as shown by the fact that the loss of tumor-suppressive activity of miR-142 stimulated cell growth and led to the induction of DLBCL [106]. miR-199 is known to downregulate inhibitor of NF-κβ, which leads to survival, proliferation, and apoptosis of lymphoma cells [89, 109]. The ubiquitin-proteasome inhibitor, bortezomib (Velcade^®^), was approved by the FDA in 2008 for treating newly diagnosed and relapsed/refractory multiple myeloma and multiple mantle cell lymphoma patients, has also shown activity in DLBCL patients (Supplementary Table 5) [110]. In addition, the expression of particular miRNAs may serve as unique biomarkers for DLBCL diagnosis, subtype classification, and outcome prediction [109].

### Other important biomarkers

Other important biomarkers in DLBCL may also have an impact on the prognosis of this disease (Supplementary Table 2) [83]. CD5 is expressed in 10% of cases of DLBCL and is typically associated with poor prognosis. CD5 expression is also commonly associated with adverse clinical prognostic characteristics including advanced age, presentation of extra-nodal involvement and diagnosis of higher-stage disease [83, 111]. CD20 is a membrane-bound protein that is widely expressed in B-cells, including in patients with DLBCL, and plays a role in the activation, differentiation and progression of the cell cycle [112]. The addition of monoclonal antibody directed against the CD20 antigen, Rituximab, to CHOP (cyclophosphamide, doxorubicin, vincristine, and prednisone) has shown to dramatically improve the survival of patients with DLBCL [37, 113]. Expression of Ki-67, a nuclear antigen expressed by cycling cells, is highly variable, ranging from 20–80% and occasionally reaching 80–100% in DLBCL cases [37, 83]. Ki-67 overexpression correlates with inferior overall survival (OS), reduced event-free survival (EFS), and poor prognosis in patients treated with R-CHOP [37]. The multifunctional type I transmembrane CD43 glycoprotein (leukosialin) is expressed in a variety of hematopoietic cells including B lymphocytes and in a variety of malignancies [114], including lymphoma, leukemia, and solid tumors, as well as in 20% of DLBCLs [37]. In DLBCL patients, CD43 expression was associated with lower complete response, OS, and EFS compared with CD43-negative DLBCL patients. The effects of CD43 were found to be significant in DLBCL patients treated with R-CHOP, but not CHOP [37, 83].

Biomarkers in DLBCL derive from a variety of classes of cellular molecules such as transcription regulators, cell cycle regulators, tumor suppressors, and necrotic factors, DNA/RNA repair regulators, and immune/inflammatory and other signaling genes; their roles in the pathogenesis of DLBCL are described in Supplementary Table 2. Genome-wide studies of associated biomarkers illustrate the complexity and heterogeneity of DLBCL. DLBCL has a high level of genomic instability; the availability of biomarkers associated with consequences of that instability provides a potential avenue for the development of novel therapeutic strategies [115].

## TREATMENT OF EARLY STAGE DLBCL

Early-stage DLBCL, usually categorized as Stage I or Stage II, accounts for 25% (up to ~40%) of all patients with DLBCL, and is known as ‘limited stage lymphoma.’ Limited stage lymphoma can be further differentiated as non-bulky, with cancerous masses having diameters of 10 centimeters or less, and bulky, with cancerous masses of 10 centimeters or greater. According to NCCN Guidelines for DLBCL, the preferred modality of treatment for Stage I and II DLBCL is immuno-chemotherapy, including combination chemotherapy, most commonly with R-CHOP typically given over a period of 3–4 months. Rituximab, the “R” in R-CHOP, is a chimeric monoclonal antibody that targets CD20, which is present on normal and most malignant B-cells [113]. Preclinical models have demonstrated that rituximab potentiates the effect of several chemotherapeutic agents [116, 117]. Compared with the CHOP regimen, R-CHOP can significantly improve complete remission rates, prolong EFS, and increase ORR and OS with a minimal increase in toxicity [118]. Both non-bulky and bulky DLBCL patients usually receive up to 6 cycles of induction chemo-immunotherapy with R-CHOP [30]. Early PET evaluation after 1 to 2 cycles of R-CHOP is less predictive of outcome than at the end of treatment scan [119–121]. At the completion of treatment, all prior positive imaging studies are repeated. If the PET-CT scan is positive, then a repeat biopsy is generally performed prior to changing the course of therapy.

An additional option for early-stage or untreated DLBCL patients would be to enroll in any of the numerous clinical trials currently available [122]. Among completed clinical trials, one of the most promising approaches to date for early-stage or untreated DLBCL assessed a combination of rituximab and epratuzumab (NCT00301821). This pilot study resulted in a 12 month OS rate of 89% [123], as well as increased rates of progression-free survival (PFS) (Supplementary Table 3). In a subsequent phase-2 study, 3-year EFS and OS rates were 70% and 80%, respectively [123]. The high survival rates and excellent safety profiles of CD20 and CD22 combination therapy represents an attractive target in B-cell malignancies, particularly in patients who are Rituximab resistant and who are not high dose chemotherapy (HDC) candidates. As CD22 is internalized when binding to the antibody, the combination of cytotoxic agents with epratuzumab can lead to improved outcomes [124]. In addition, a more recent clinical trial demonstrated that chemotherapy, combined with lenalidomide with or without rituximab, did not result in adverse effects or toxicity [122, 125].

In rare cases, radiotherapy alone is the only option of treatment for early-stage DLBCL patients who are unable to tolerate chemotherapy, especially elderly, frail, or co-morbid patients [126]. Multiple randomized and retrospective studies have shown that consolidation RT significantly reduces the risk of recurrence of the disease after CHOP therapy in stage I-II DLBCL [127]. Optimisation of disease management thus reducing acute and late side effects is important as many DLBCL patients are long-term survivors [128]. A study by Phan et al. (2010) [129] found that OS and PFS were significantly improved among patients who received consolidation radiation treatment after undergoing R-CHOP therapy. Patients treated with three CHOP plus RT cycles had significantly better PFS (*P* value = 0.03) and OS (*P* value = 0.02) than those treated with CHOP alone [130]. The five-year estimates of PFS for patients receiving RT and CHOP therapy vs. patients who received CHOP therapy alone were 77% vs. 64%, respectively [131].

## TREATMENT OF ADVANCED STAGE DLBCL

Advanced stage DLBCL, stage III/IV, accounts for 75% of DLBCL patients with 5-year survival rates for R-CHOP of 50–55% [132]. The question of dose-dense and dose-intense chemotherapy with rituximab has been addressed previously in several trials demonstrating no additional benefit over R-CHOP [133–136]. In a randomized trial, the GELA group compared, R-ACVBP (rituximab plus doxorubicin, cyclophosphamide, vindesine, bleomycin, prednisone) intensified chemotherapy with R-CHOP in low IPI DLBCL [136]. R-ACVBP showed superior 3-year EFS (81% Vs. 67%) and OS (92% vs. 84%) rates compared to R-CHOP [136]. However, R-ACVBP was associated with severe toxicity; therefore, it is not frequently used in clinical practice. HDC followed by autologous stem cell transplant (ASCT) as the first-line treatment of advanced-stage DLBCL has remained questionable in several clinical trials. ASCT has shown substantial benefits to the patient [137]. Randomized trials have compared R-chemotherapy, followed by HDC and ASCT versus R-chemotherapy alone for high IPI patients. Some trials demonstrated favorable PFS rates for HDC and ASCT with no additional benefit for OS [138, 139]. Therefore, the standard first-line therapy for advanced-stage patients remains six cycles of R-CHOP-21 with interim scan after 2 to 4 cycles. If the patient responds to therapy, R-CHOP is continued for a total of 6 cycles, or the patient is enrolled in a clinical trial. At the completion of treatment, all positive studies will be repeated with a PET/CT scan. A re-biopsy must be conducted before the course of treatment is modified. If the PET/CT scan indicates, a PR or no response (progressive disease), second-line therapy is pursued.

An intergroup, phase III study; Alliance/CALGB 50303 (NCT00118209), compared standard rituximab, cyclophosphamide, doxorubicin, vincristine, and prednisone (R-CHOP) with dose-adjusted etoposide, prednisone, vincristine, cyclophosphamide, doxorubicin, and rituximab (DA-EPOCH-R) as frontline therapy for diffuse large B-cell lymphoma [140]. In the efficacy analysis, R-CHOP and DA-EPOCH-R showed no statistically significant differences in overall response rate (ORR) or other outcomes (Supplementary Table 3). The EFS was similar throughout the median follow-up of 5 years (hazard ratio [HR], 1.14; 95% confidence interval [CI], 0.82–1.61; *P* value = 0.4386). Overall survival was also similar in both treatment groups (HR, 1.18; 95% CI, 0.79–1.77; *P* value = 0.42) [140].

Obinutuzumab (G) is a type II glycoengineered, monoclonal antibody anti-CD20 (proves more potent antibody-dependent cellular toxicity and greater apoptosis induction than rituximab) (Supplementary Table 6) [141, 142]. GOYA was a phase III randomized trial comparing G-CHOP to R-CHOP in patients with previously untreated advanced-stage DLBCL [143]. The GOYA trial enrolled 1,418 patients with treatment-naïve, CD20-positive DLBCL. For eight 21-day cycles, patients were randomly allocated to open-label care with obinutuzumab (*n* = 706) or rituximab (*n* = 712) in conjunction with standard CHOP for 6 or 8 cycles. The primary outcome was progression-free survival (PFS) assessed by the investigator [144], after a 29-month median follow-up, there was no difference between R-CHOP and G-CHOP in PFS assessed by the investigator (HR, 0.92; 95% CI, 0.76–1.11; *P* value = 0.3868). Disease-free survival, EFS, and time to next treatment in both treatment groups were also identical. DLBCL cell-of-origin (COO) subgroups also analyzed PFS: germinal-center B cell-like (GCB), activated B cell-like (ABC), and unclassified subgroups (exploratory analysis) [145]. GCB subtype is associated with a better outcome than the ABC or unclassified subtypes [143]. A recent study was conducted to evaluate ibrutinib, and R-CHOP in stage II to IV in previously untreated non-GCB DLBCL patients, the primary endpoint was event-free survival (EFS) in the intent-to-treat (ITT) population, and secondary endpoints included progression-free survival (PFS), overall survival (OS), and safety. The study did not reach its primary endpoint in the population of ITT or ABC. However, ibrutinib plus R-CHOP improved EFS, PFS, and OS with manageable protection in patients younger than 60 years of age, but in patients 60 years of age or older it was associated with increased toxicity, leading to poor R-CHOP administration and worse outcomes [146].

## TREATMENT OF RELAPSED/REFRACTORY DLBCL

Despite advancements in DLBCL treatment over the last decade, there are still up to 40% of primarily refractory patients or experience short-term relapses, mostly occurring within 1–2 years of remission. The first choice for relapsed/refractory patients is to determine whether patients are transplant eligible or no. If patients are transplant eligible, then salvage chemotherapy is followed by ASCT, provided patients to respond to treatment. Salvage regimens for those patients include DHAP (dexamethasone, cisplatin, cytarabine) ± rituximab, GDP (gemcitabine, dexamethasone, cisplatin) ± rituximab, ESHAP (etoposide, methylprednisolone, cytarabine, cisplatin) ± rituximab, or ICE (ifosfamide, carboplatin, etoposide) ± rituximab [30] and these regimes have a similar outcome. However, GDP ± rituximab is less toxic than DHAP ± rituximab [147]. DHAP ± rituximab has been shown to improve the survival in the GCB-type, although this needs validation. Patients who are not candidates for HDC may be treated with other salvage regimens including bendamustine ± rituximab, CEPP (cyclophosphamide, etoposide, prednisone, procarbazine) ± rituximab, CEOP (cyclophosphamide, etoposide, vincristine, prednisone) ± rituximab, DA-EPOCH, GEMOX ± rituximab, GDP ± rituximab, or lenalidomide ± rituximab (Supplementary Table 3) [38].

If patients have no response to second-line therapy, they would then be considered for a clinical trial or palliative RT. Supplementary Table 3 lists recent clinical trials for relapsed/refractory DLBCL. A more recent clinical trial (NCT00869999) using mTOR inhibitors, everolimus or temsirolimus (CC1-779) in combination with rituximab, demonstrated complete responses in 12.5% and 28.1% of patients on the study, respectively, and increased the OS rate to 37% at a median follow-up of 12 months. In general, everolimus was well tolerated and demonstrated activity in relapsed DLBCL patients in combination with rituximab [148, 149]. Among all completed trials of relapse/refractory DLBCL patients, ibrutinib, a Bruton’s Tyrosine Kinase inhibitor (upstream of NF-κB signaling pathway), has provided an excellent example of precision medicine. In this trial, ibrutinib showed complete or partial responses in 37% (14 of 38) of patients with ABC DLBCL but only 5% (1 of 20) of patients with GCB DLBCL [150]. Conversely, bortezomib, despite inhibiting a key B-cell activation molecule, showed varied responses with significant benefit in relapsed/refractory ABC-DLBCL [151] but no clinical benefit in newly diagnosed non-GCB DLBCL patients [152, 153]. Results from the Pyramid Trial (NCT00931918), the addition of bortezomib to R-CHOP (VR-CHOP) in patients with previously untreated non-GCB DLBCL was evaluated for the efficacy and safety, suggest no significant efficacy advantage with the addition of bortezomib to R-CHOP in patients (maybe due to lack of bortezomib effect or patient selection or misclassification [154]. This suggests that compounds inhibiting different steps of signaling pathways have different clinical efficacy.

**Figure 3:**
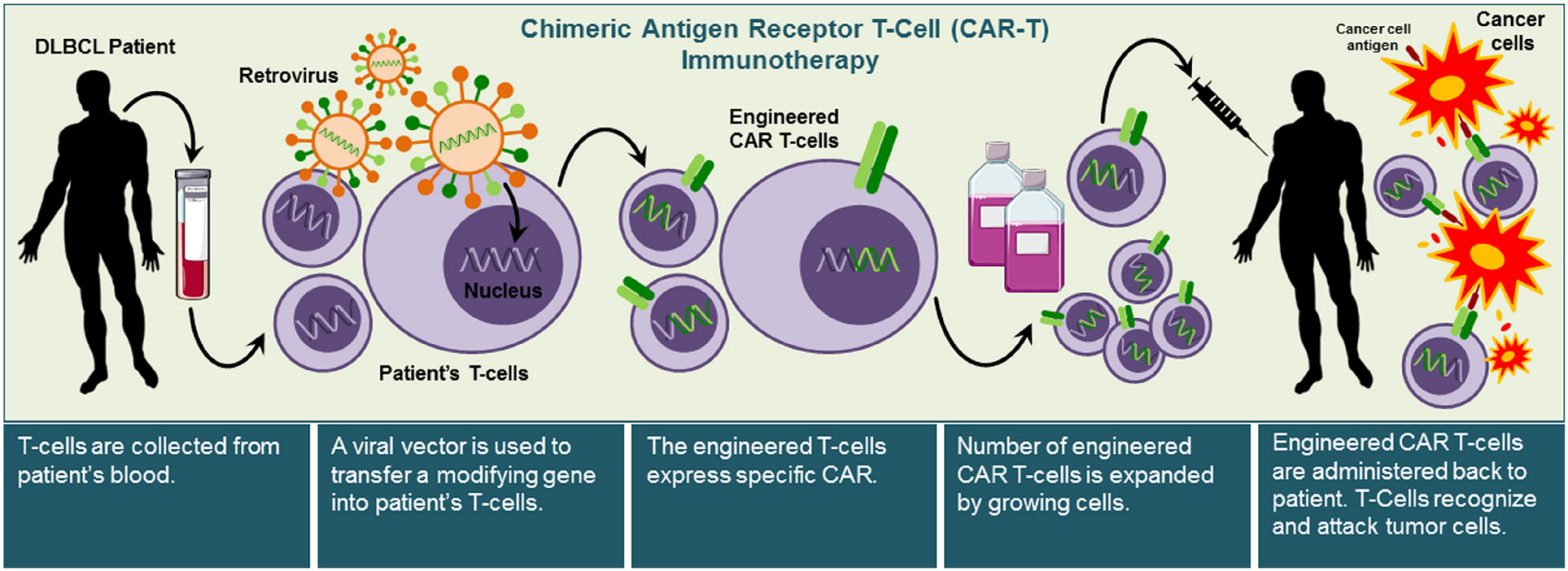
Illustration of chimeric antigen receptor T-cell (CAR-T) immunotherapy approach. CAR-T immunotherapy approach allows the use of the patient’s T-cells to attack cancer cells. CAR-T immunotherapy requires collecting patient’s immune cells and modifying them to express specific CARs that recognize specific cancer antigens by transfecting T- cells with viral vectors. Once T cells are successfully engineered to express the antigen-specific CAR, they are expanded and then re-administered into the patient to recognize and kill cancer cells.

Chimeric antigen receptor T cell (CAR-T) immunotherapy is a novel approach for patients with refractory aggressive B-cell lymphoma. Briefly, T cells are engineered for the identification and destruction of cancer cells. The CAR-T treatment removes a person's T cells from the body, genetically modifies the specific cells using a retrovirus vector to introduce new genes, and then returns the cells to the patient, where they can attack cancer cells (Figure 3) [32]. These trials are enrolling recurrent/refractory DLBCL patients 18 to 70 years old (Supplementary Table 4).

Phase 2a JULIET trial study adds mounting evidence that chimeric antigen receptor (CAR) T-cell therapy (KYMRIAH^®^, tisagenlecleucel) targets and removes CD19-expressing B cells and has shown to be effective against B-cell lymphoma. A total of 93 patients were infused and included in the efficacy assessment [155]. The overall response rate was 52%, and 40% of the patients had complete responses, and 12% had partial responses. The rate of relapse-free survival was estimated at 65% at 12 months after the initial response (79% among patients with complete response). Cytokine release syndrome (22%), neurological disorders (12%), cytopenias lasting more than 28 days (32%), infections (20%), and febrile neutropenia (14%) were the most frequent grade 3 or 4 adverse effects of particular concern. Tisagenlecleucel, cytokine-release syndrome or cerebral edema were not attributed to death [155].

Results of ZUMA clinical trial phase 2 were presented in ASH (2017) with one year of follow-up, Among the 111 patients who were enrolled, Axicel was manufactured successfully at 110 (99%) and administered at 101 (91%). The objective response rate was 82%, and the full response rate was 54%. For a 15.4-month median follow-up, 42% of patients continued to have a response; 40% continuing to have a complete response. At 18 months, the overall survival rate was 52% [156].

Nonetheless, SCHOLAR-1 showed disappointing results in refractory DLBCL patients, suggesting a need for more successful therapies for these patients. The objective response rate for patients with refractory DLBCL was 26% (complete response rate, 7%) to the next therapy line, and the overall median survival was 6.3 months. Twenty percent of patients were alive at two years [157].

In responsive patients, the anti-CD19 CAR T-cell therapy achieved complete and durable responses in aggressive DLBCL despite translocations of MYC, BCL2, and BCL6 and their overexpression (Supplementary Table 4) [158].

FDA has been approved CAR-T therapy (Clinical trials of chimeric genetically modified T cells for CD19 by Kite Pharma) for diffuse large B-cell lymphoma (DLBCL), high-grade B-cell lymphoma, and DLBCL from follicular lymphoma (transformed follicular lymphoma, or TFL), not otherwise specified [159]. A risk assessment and mitigation strategy is approved, however, and risks include cytokine release syndrome (CRS), viral reactivation, persistent cytopenias, and neurological toxicity [160].

Since DLBCL is already curable in 60–65% of patient with R-CHOP, the CAR-T approach may become one among many salvage options over the next several years. CAR-T therapy is curative for leukemia in children (ALL) and has the potential to become an once-in-a-lifetime treatment due to long-term durability with durations of remission, but it is premature to say [161]. CAR-T therapy may replace existing therapies for ALL, but for DLBCL related B cell malignancies, R-CHOP will remain the standard therapy.

## PROSPECTIVE CLINICAL TRIALS

Several drug classes and strategies that are effective in other cancers will likely become subjects of future prospective clinical trials for the treatment of DLBCL patients since the clinical evidence has shown its efficacy in other cancers. Among these are immune checkpoint inhibitors and CTLA blockade.

Checkpoint inhibitors work by targeting receptors that serve as brakes on the immune response. Checkpoint inhibitors may be used to activate or improve pre-existing anti-cancer immune responses by blocking certain inhibitory molecules or by activating stimulating molecules. Programmed Cell death 1 (PD-1) receptor is the target for pembrolizumab and related drugs. PD-1 down-regulates the immune system and reduces inflammatory behavior in T cells [162]. Drugs that block PD-1 activity can activate the immune system, permitting immune T cells to attack tumors. Pembrolizumab has been used to treat certain kinds of cancer with varying degrees of effectiveness. Pembrolizumab has shown promising results in metastatic melanoma, metastatic non-small cell lung cancer in several clinical trials, and most recently approved for the treatment of squamous cell cancer in the head and neck (HNSCC) [163].

In 2014 the FDA approved pembrolizumab for the treatment of metastatic melanoma. In 2016, pembrolizumab was approved for treating HNSCC after a completed trial analysis showed a 16% objective response rate and a complete response rate of 5% with responses lasting for more than 6 months in 82% of patients [164]. The FDA has also approved pembrolizumab for the treatment of relapsed/refractory adult and pediatric classical HL patients (cHL). The findings of a phase 1b cHL relapsed/refractory study (KEYNOTE-013) were positive and comparable to nivolumab with PFS levels of 69% and 46%, respectively, at 24 weeks and 1 year [165, 166]. In a subsequent phase II trial (KEYNOTE-087), the activity of pembrolizumab was evaluated in cHL patients who had failed either ASCT, brentuximab vedotin, or both. The ORR across entire cohorts was 69% [167].

In a multicohort phase 1b trial of relapsed/refractory primary mediastinal large cell lymphoma (PMBCL) patients, pembrolizumab showed promising anti-tumor activity with a tolerable safety profile of 41% [168]. These results suggest that checkpoint inhibitors like pembrolizumab would be effective in the treatment of DLBCL.

The addition of an immune checkpoint inhibitor to standard R-CHOP therapy for DLBCL would be expected to activate stimulatory molecules of the immune system to attack tumors. Naturally, it is also of interest to use PD-1 blockade early in the course of treatment, to improve cure levels in high-risk patients or to reduce the toxicity of treatment in patients at lower risk [169].

In theory, PD-1 blockade, alone or in combination, could be used in frontline therapy or early salvage, and those studies are highly anticipated [169]. In this regard, a trial is currently underway (NCT02362997) testing repeated dosing of pembrolizumab in patients who have undergone ASCT for Relapsed Refractory cHL or DLBCL [170]. The therapeutic regimen calls for 200 mg intravenous pembrolizumab every three weeks for up to 8 cycles, beginning within a few weeks of ASCT. The results of this on-going trial are not yet available.

Another area of interest for a prospective clinical trial to treat DLBCL is to combine pembrolizumab, a PD-1 inhibitor, with ipilimumab, an antibody against cytotoxic T-lymphocyte-associated antigen 4 (CTLA-4). This drug combination has shown prolonged overall survival in patients with advanced melanoma in a Phase 2 trial (NCT01024231), with 53% of patients having an objective response and tumor reduction of 80% or more. Additionally, even maximum doses were not associated with increased toxicity [171].

CTLA-4 is a negative regulator of T cell activation. CTLA-4 blockade is intended to release anti-cancer T cells permitting them to attack tumor cells. Several clinical trials of drugs that block CTLA-4 have been conducted in advanced melanoma with concurrent radiotherapy. In one particular Phase 2 study, patients treated with ipilimumab in combination with radiotherapy showed improved survival and higher complete response rates than patients treated with ipilimumab only. Median overall survival in the ipilimumab-radiotherapy (Ipi-RT) arm (19 months) was significantly higher than in patients treated with ipilimumab alone (10 months) (*P* value = 0.01). CR levels in the Ipi-RT group (25.7%) were significantly higher than in patients given ipilimumab alone (6.5%) (*P* value = 0.04) [172]. In addition, OR rates in the groups were 37.1% vs. 19.4% (*P* value = 0.11). No increase in toxicities was observed in the Ipi-RT group compared with ipilimumab alone [172]. Combination therapy with ipilimumab and ionizing radiation for the treatment of Metastatic Non-small cell lung cancer has also shown positive results in Phase II clinical trials (NCT02221739).

Combination therapy with two checkpoint inhibitors, epacadostat, and pembrolizumab has shown a 56 percent overall response rate in treatment-naïve patients with advanced melanoma and a 75 percent disease control rate [173]. Based on these results, enrollment in tumor-specific cohorts is ongoing in phase 2 of this study, and a phase 3 trial for advanced melanoma in treatment-naive patients has been initiated (NCT02752074). The success of epacadostat and pembrolizumab in melanoma suggests that this combination might be appropriate for a clinical trial for use in DLBCL treatment.

The combination of nivolumab, a checkpoint inhibitor that targets PD-1, and ipilimumab, the CTLA-4 blockade drug in the treatment of naïve advanced melanoma, has shown significantly increased progression-free survival compared with ipilimumab alone. Median follow up at 20.7 months, progression-free survival was significantly longer when these drugs were combined. The reduction of risk of progression or death was 58% for patients receiving the combination versus 45% for patients treated with ipilimumab alone. The median PFS for the combination was 11.5 months, 6.9 months for nivolumab alone and 2.9 months for ipilimumab alone [174]. In CheckMate 039, trial patients with relapsed/refractory hematologic malignancies were treated with Nivolumab and ipilimumab combination. Promising results were observed for HL patients achieving ORR of 74%. DLBCL patients showed ORR of 20% after 11.4 months of follow-up [175]. The study showed a safety and efficacy profile similar to the previous one that included only Nivolumab in HL, NHL, and MM. Long-term results in a large patient cohort will provide a better understanding of the role of this immune checkpoint blockade in DLBCL and other hematologic malignancies.

## NOVEL DRUGS FOR DLBCL

Approximately 30–40% of patients treated with R-CHOP or a similar inhibitor relapse after completing therapy, and often develop resistance to rituximab. Overall, 60–65% of DLBCL patients are cured by R-CHOP [96, 176]. Drug resistance can be classified into three categories: genetic resistance, resistance to chemotherapy, and tumor microenvironment (TME) cell adhesion-mediated drug resistance [96]. For patients who become rituximab resistant, several clinical trials are underway to assess drugs that target specific pathways, including NF-κB and PI3K/AKT/mTORC, Bruton’s tyrosine kinase (BTK), Spleen tyrosine kinase (STK), Enhancer of zeste hormone 2 (EZH2), phosphoinositide-3-kinase (PI3K), mammalian target of rapamycin (mTOR), Janus Kinases (JAK), and B-cell lymphoma proteins (BCL) [96]. An extensive list of novel drugs targeting these pathways their pharmacological profiles are provided in Supplementary Tables 5 and 6, respectively. We also provide a schematic diagram describing the molecular targets for the therapeutic agents used for DLBCL (Figure 2).

### BCL2 inhibitors

BCL-2 is associated with the regulation of apoptosis [177]. Despite frequent BCL-2 overexpression, Venetoclax, a BCL-2 inhibitor, has limited activity in DLBCL. Since the B cell receptor (BCR) pathway is constitutively activated in both ABC and GCB DLBCL, Sasi BK et al. (2019) [178] showed SYK or BTK inhibition synergistically enhances the responsiveness of venetoclax in both BCL-2-positive DLBCL cell lines *in vitro* and *in vivo* in the xenograft mouse model. They also showed that BCR-dependent GCB DLBCL cells are characterized by a deficiency of the BCR pathway’s phosphatase SHP1 [178].

B-cell receptor inhibition (BCR) signaling pathway is a promising therapeutic strategy for multiple B-cell malignancies. However, we don’t know the role of inhibition of BCR in DLBCL. A study by Bojarczuk K et al. (2019) [59] shows that a combination of PI3Kα/δ inhibitor (copanlisib) and BCL2 inhibitor (venetoclax) extended the median survival of treated mice significantly longer than single-agent venetoclax. This combination set the stage for clinical evaluation of copanlisib and venetoclax in patients with genetically defined BCR-dependent DLBCLs.

Venetoclax (ABT199) was approved for CLL 17p deletion in 2016; it has shown positive activity in relapsed/refractory DLBCL with an overall response rate (ORR) of 33% and a complete response rate (CR) of 11% with 600 mg as the max dose (NCT01328626) [179]. An issue observed in cell lines treated with ABT199 is the compensatory upregulation of MCL-1. If ABT199 is paired with AT7519, a multi-CDK inhibitor (completed phase II clinical trials (NCT01652144) for MCL, CLL, and has been combined with Bortezomib for MLL treatment), the treatment produces indirect downregulation of MCL-1. The combination inhibits particular CDK pathways, e.g., CDK4 (Supplementary Table 2) [180, 181]. Thus, the combination of ABT199 and AT7519 might be effective for treating some unresponsive or relapsed DLBCL patients.

Drugs such as ABT-737 bind to BCL-2 and BCL-xL and cause suppressed tumor growth *in vivo* [96]. Navitoclax (ABT-263), another oral BCL-2 and BCL-xL inhibitor [96, 182], exhibited an ORR of 21.7% with a median PFS rate of 14.9 months in a phase I study of NHL relapse patients [96]. However, this navitoclax exhibits notable adverse events, including thrombocytopenia and lymphopenia [182].

### BCL6 inhibitors

For B-cells, BCL-6 is the master transcriptional regulator of germinal center cells and is responsible for the regulation of cell growth, metabolism, and survival [183]. The upregulation of BCL-6 correlates with poor response rates and rituximab resistance among GCB-DLBCL cases. BCL-6 upregulates SYK activity indirectly by repressing PTPROt, a protein regulating SYK. The increase in BCL-6 also increases the activation of SYK and leads to an anti-apoptotic effect [184]. BCL6 loss of function, mediated by delivery of shRNA or peptide inhibitors, can kill DLBCL cells, showing that BCL6 is necessary for lymphoma cell survival and could represent an excellent therapeutic target [185, 186]. Recent studies have shown that HSP90 forms a BCL6 complex and inhibits HSP90 with the drug PU-H71, a purine scaffold HSP90 inhibitor destabilizes BCL6 and selectively destroys *in vitro* and *in vivo* BCL6-positive DLBCL cells [73, 185].

BCL-6 inhibitor, 79-6, directly antagonizes protein function by binding to the lateral groove, which leads to the inhibition of the BTB domain, and consequently disrupts proteins that co-repress apoptosis and block protein-protein interaction pathways [96, 183]. 79-6 also disrupts transcriptional BCL6 complexes and indirectly reactivates target BCL6 genes. BCL6 mediated repression of the ATR gene has been suggested to contribute to the lymphomagenic actions of BCL6 and is dependent on the BCL6 lateral groove [187].

### BTK inhibitors

Bruton’s tyrosine kinase (BTK), a member of the tyrosine-protein family, links BCR signaling to various downstream pathways, including AKT, MAP kinase, and NF-κB activation signaling pathways together with calcium release [183]. It has been shown that mutated BTK is associated with loss of B-cell function [183, 188]. BTK is also responsible for the survival of CD79 mutated ABC-DLBCL cells [189]. Ibrutinib (PCI-32765), an irreversible oral BTK inhibitor that binds to the cysteine-481 (Cys481) residue within a BTK activation site, prevents Tyr223 phosphorylation [183, 188, 190]. A phase I study of combination treatment with ibrutinib (PCI-32765) and chemotherapy (R-ICE) resulted in an ORR of 60% in relapsed NHL and 17% in DLBC [191] (see Supplementary Table 3). A phase II trial study of ibrutinib in ABC- and GCB- DLBCL subtypes resulted in an ORR of 40% and 5%, respectively [183, 190, 192]. In a phase I trial of ONO-4059 (NCT01659255), a BTK inhibitor that acts by inducing classical apoptosis, the ORR was 35% in ABC-DLBCL patients [96, 193].

### mTOR inhibitors

The mTOR signaling pathway mediates growth signaling that originates from PI3K. Signaling through the PI3K pathway can be modified through mutation or amplification of AKT. Moreover, mTOR activation *via* AKT induces the proliferation and survival of cells [188]. The mTOR pathway aids in the regulation of protein translation, cell growth, and metabolism. Hyperactivation of the mTOR pathway can potentially lead to the suppression of cell autophagy machinery and subsequent cancer development [191]. mTOR proteins can be divided into two subgroups, mTORC1 and mTORC2 [191]. mTORC1 regulates mRNA transcription through activation of S6K1 and 4EBP1. MTORC2 controls the transportation of nutrients and amino acids, stimulates PKC-phagy AKT and is immune to rapamycin inhibitor mTORC1 [191]. DLBCL patients treated with temsirolimus presented a PFS rate of 2.6 months, an ORR rate of 28.1%, and a complete response rate of 12.5% in one clinical trial. Following completion of R-CHOP treatment, DLBCL patients achieved an ORR of 30% with maintenance medication; the oral rapamycin analog everolimus, an alternative to temsirolimus. In phase III clinical trial, everolimus was compared to placebo in DLBCL patients receiving first-line treatment with R-CHOP (Supplementary Table 4). The results of this study are pending. Temsirolimus and everolimus have similar toxicity profiles, and both have shown clinical responses in various NHL subtypes. However, the low response rates of these drugs as single agents suggest that combination studies are needed [189, 193].

### PI3K inhibitors

PI3K and its components assist in the regulation of cell growth, metabolism, migration, and survival [188]. PI3K is upregulated in GCB-DLBCL tumors, and mutation or overexpression of PI3K leads to the activation of the mTOR pathway and tumorigenesis [96, 191, 194]. The mTOR pathway is involved in growth signaling and cell metabolism. Furthermore, activation of the PI3K/AKT/mTOR pathway results in gene expressions, loss of PTEN, or constitutive activation of upstream regulatory pathways [183]. PI3K inhibitors, including LY294002 and idelalisib, an FDA-approved selective PI3K inhibitor, have shown promise in treating GCB-DLBCL [96, 195]. Both compounds are currently under clinical evaluation for DLBCL [21].

Other studies targeting the PI3K pathway have been promising. B-cell lymphoma patients treated with DA-EPOCH-R and R-HCVAD/MD (rituximab, hyperfractionated cyclophosphamide, vincristine, doxorubicin, and dexamethasone alternating with rituximab, high-dose methotrexate, and cytarabine) had a higher CR compared to patients treated with R-CHOP and ABVD (doxorubicin, bleomycin, vinblastine, dacarbazine) [196]. Targeting the PI3K pathway could show an increase in recovery rates amongst relapsed and refractory patients. In patients with relapsed and refractory Waldenstrom Macroglobulinemia (another non-Hodgkin Lymphoma) who were treated with everolimus in combination with bortezomib and rituximab, the ORR in phase I and II studies combined was 89% [197]. Efficacy and safety of the PIK3 inhibitor copanlisib were evaluated in 67 patients with relapsed/refractory DLBCL of ABC and GCB in phase II study. This trial demonstrated a manageable safety profile in patients with relapsed/refractory DLBCL and a numerically higher response rate in ABC vs. GCB DLBCL patients. However, the difference was not statistically significant. However, data suggest that despite the worse prognosis as expected, ABC DLBCL may respond preferentially to inhibition of PI3K signaling [198].

### PKC inhibitors

PKC-I is a serine/threonine kinase that aids in the propagation of BCR signaling and activation of the NF-κB pathway [199]. PKC-I plays an essential role in biological processes related to signal transduction, cell proliferation, and apoptosis. In addition, it also acts as a key component of the BCR signaling pathway and is involved in the normal function of B-cells [177]. Overexpression of PKC has been associated with poor outcomes for DLBCL patients [96]. Two PKC inhibitors are currently in use or trials for DLBCL. Enzastaurin is an oral PKC-β inhibitor that is well-tolerated in newly diagnosed and relapsed/refractory DLBCL patients [200], and the second PCK inhibitor, sotrastaurin, is currently in clinical development for DLBCL [201].

### SYK inhibitors

Most B-cell malignancies express the B-cell receptor (BCR) [202]. Spleen tyrosine kinase (Syk) initiates and amplifies the BCR signal, Syk inhibition induces apoptosis in B-cell lines and primary tumors [203, 204]. A phase 1/2 clinical trial of the first commercially available oral Syk inhibitor, fostamatinib disodium, was performed in 68 patients with recurrent B-cell non-Hodgkin lymphoma (B-NHL). The results of this study indicate that disrupting BCR-induced signaling by inhibiting Syk represents a novel and successful therapeutic strategy for NHL since patients’ median progression-free survival was 4.2 months [203].

Another phase II clinical trial was performed to determine the efficacy of Fostamatinib in patients with GCB and ABC persistent or refractory DLBCL and COO cell signatures [205]. This trial was to further prove the clinical benefits of Fostamatinib in subtypes of DLBCL only, but due to poor schedule and doses, GCB and intermediate patients clinically benefited; none of the clinically benefited patients had ABC genotype [205].

A selective Syk inhibitor, Entospletinib (GS-9973), is an oral, was evaluated in a Phase II study with 43 relapsed or refractory DLBCL patients [206]. No patient achieved a complete or partial response; however, 12% had stable disease, and PFS at 16 weeks was 3.6%, demonstrated limited activity in patients [206].

Cerdulatinib is a dual inhibitor of Syk and JAK 1/3 and *in vitro* studies 9 have been shown to have efficacy against DLBCL. Cerdulatinib is an inhibitor of oral Kinase against Syk and JAK [207]. Cerdulatinib mediated apoptosis in both ABC and GCB lymphoma cell lines, and cell cycle arrest. Cerdulatinib suppressed the signaling of JAK / STAT and BCR in primary DLBCL and non-GCB tumor cells [208].

The screening and optimization of a novel series of heteroaromatic Syk inhibitors resulted in the discovery of TAK-659, a dual Syk and FLT3 inhibitor [209]. A clinical trial of TAK-659 included 5 GCB, 2 ABC DLBCL subtypes and 10 lymphomas patients (7 had follicular lymphoma). All 7 DLBCL patients respond to treatment, out of them 3 achieved PR. Thus, TAK-659 seems acceptable for further studies [210, 211].

### MALT1 inhibitors

MALT-1 is essential for the activation and regulation of NF-κB and BCR stimulation [96, 189]. MALT1 is required for the survival of B-cells in ABC-DLBCL and acts as a protein scaffold that recruits other critical signaling molecules, such as TRAF6, caspase 8, and A20, to the CARD11-BCL10-MALT1 (CBM) complex [189]. Z-VRPR-FMK is an irreversible, peptide-based MALT1 inhibitor that was first designed as an inhibitor of meta-caspases in plants. At low concentrations, Z-VRPR-FMK can block cleavage activation [212]. Although Z-VRPR-FMK is useful for research, it has not been suitable for clinical use due to its size, charge, and low permeability [213]. A comparison of the effects of the MALT1 phenothiazine, thioridazine and mepazine inhibitors on DLBCL cell lines showed that mepazine was the most effective cellular MALT1 inhibitor and decreased MALT1 activity by 75% in all ABC-DLBCL cell lines tested [189, 214]. Mepazine does not directly affect substrate binding to the catalytic center of MALT1 in a competitive manner but instead acts as a non-competitive and reversible inhibitor. Moreover, the S-enantiomer of mepazine, s-mepazine, in combination with the BTK inhibitor ibrutinib, also induced a reduction in MALT1 activity in ABC-DLBCL cell lines [189].

### JAK inhibitors

The inhibition of JAK2 blocks the activation of STAT3 and STAT1. Activation of the JAK1/JAK2-STAT3 and JAK1/JAK2-STAT1 cascades has been shown to upregulate survival factors. STAT3 activation in ABC-DLBCL patients treated with R-CHOP also is associated with poor OS [215]. Moreover, inhibiting STAT3 activity has been shown to sensitize resistant B-cell NHL cells to chemotherapeutic cytotoxic drugs. Inactivation of STAT3 in ABC-DLBCL induces apoptosis and reduces cell proliferation *in vitro* [96]. The JAK1/JAK2 inhibitor Fedratinib (TG101348) reduces the growth and phosphorylation of STAT1 and STAT3 in ABC-DLBCL cells [96]. JAK inhibitors have been associated with multiple side effects. Currently, Pacritinib (SB1518) is the only JAK-2 inhibitor under clinical development [188].

### NF-κB inhibitors

The upregulation of NF-κB is a distinct characteristic of ABC-DLBCL [92]. NF-κB is a transcription factor that controls multiple cellular functions linked to tumor development, such as inflammation, cytokine secretion, and cellular proliferation [91]. Interruption of NF-κB signaling with an IκB super repressor or with a small molecule inhibitor of IKKβ induces apoptosis in ABC DLBCL but not GCB DLBCL cell lines [92, 216]. A biological consequence of NF-κB signaling in ABC DLBCL is to propel the malignant cell forward toward the plasma cell stage of differentiation. An NF-κB target in ABC DLBCL is IRF4 [216], a key transcription factor that drives plasmacytic differentiation [217, 218]. NF-κB upregulation of IRF-4 is characteristic of ABC-DLBCL [91].

Previously published work provides a rationale for the design of therapies targeting the alternative NF-kB pathway in a fraction of DLBCL patients and suggests that for those human DLBCLs that display both canonical and alternative NF-kB mutations [88, 90], both alternative or canonical of NF-kB signaling may be required for therapeutic intervention, as recently demonstrated for multiple myeloma [219]. A lymphopanel of 34 genes was designed based on published papers and whole exome sequencing (WES) of relapsed/refractory DLBCL patients. In Next-generation sequencing (NGS), NF-κB genes were found mutated frequently, followed by JAK/STAT pathway genes [220].

Bortezomib or carfilzomib are NF-κB inhibitors that prohibit the activation of the IκBα protein, and thus, stimulate cell cycle arrest and mitochondrial-dependent apoptosis [96]. Although bortezomib has shown no activity as a single agent, in a study of 49 relapsed ABC- and GCB-DLBCL patients, ABC-DLBCL patients treated with bortezomib plus DA-EPOCH had a higher response rate (83% vs. 13%) and higher overall survival (10.8 months vs. 3.5 months) than GCB-DLBCL patients [96]. Additional studies to compare the use of R-CHOP with or without bortezomib in newly diagnosed and refractory/relapsed DLBCL patients are ongoing (Supplementary Table 4) [96].

### STAT inhibitors

Signal transducer and activator of transcription (STAT) family proteins are responsible for regulating cellular activities, including proliferation and survival. Elevated STAT3 expression is predominantly found in ABC-DLBCL cell lines [96, 183]. STAT3 is a challenging therapeutic target because it lacks endogenous enzymatic activity [183]. Ruxolitinib, an oral inhibitor of JAK1 and JAK2, blocks phosphorylation of STAT1 and STAT3. Ruxolitinib is undergoing studies in relapsed and refractory ABC-DLBCL [96]. Pacritinib is an oral JAK2 inhibitor that also has specific *in vitro* activity against STAT3. In a phase 1 study, Pacritinib showed favorable results in relapsed or refractory lymphoma [183].

### EZH2 inhibitors

Zeste homolog enhancer 2 (EZH2) is a histone-lysine N-methyltransferase which is involved in DNA methylation and transcriptional repression [221]. It is associated explicitly with polycomb-repressive complex 2 (PRC2), the addition of methyl groups to PRC2 Lys27 aides in transcriptional suppression through chromatin remodeling. Mutations within EZH2, Y641F, and EZH2Y641F can cause increased tri-methylation of H3K27 in GCB-DLBCL [194]. EZH2 inhibitors such as CPI-360, GsK-126, and E7438 (EPZ-6438) work by selectively targeting the mutant form of EZH2. Studies are underway about the impact of E7438 on newly diagnosed DLBCL patients [194]. At the biennial meeting of the International Conference on Malignant Lymphoma (ICML) in Lugano, Switzerland (2017): Phase II Tazemetostat Trial (EPZ-6438) demonstrated an objective response rate of 29% in DLBCL with EZH2 mutation and 15% in DLBCL with wild-type EZH2 [222].

### AKT inhibitors

AKT is a serine/threonine kinase that is activated in direct response to PI3K. AKT is also the most commonly involved PI3K effector in cancer [223]. When activated, AKT can promote growth, resistance to apoptosis, and proliferation, which results in mTOR stimulation. AKT also inhibits phosphorylates and PRAS40 which adversely regulates mTORC1 (140). High levels of AKT protein in DLBCL are related to poor prognosis [224]. The most commonly described AKT inhibitor was perifosine, an oral AKT inhibitor. Effectiveness was predominantly demonstrated in large tumors and multiple myeloma [186, 225]. An allosteric AKT inhibitor, MK2206, has completed clinical and drug trials, and safety and efficacy data are available [224]. Additional clinical trials of MK2206 are currently recruiting patients with aggressive lymphomas, including DLBCL [226].

## CONCLUSIONS AND FUTURE DIRECTIONS

Over the past decade, the survival rate of patients diagnosed with DLBCL, which is the most commonly occurring NHL, has improved markedly with the implementation of the R-CHOP regimen. Nevertheless, the prognosis for 30–40% of DLBCL patients who fail R-CHOP treatment remains grim. These patients are still a challenge for the research and clinical community, and our continuing inability to treat them underscores the heterogeneity and complexity of DLBCL. This complexity is driven by chromosomal abnormalities, germline and somatic mutations, aberrations in multiple signaling pathways, and aberrant tumor microenvironment homeostasis. Improving our current understanding of the complexity of DLBCL begins with the development of precise diagnostic techniques involving biomarkers that allow for the differentiation of specific subtypes as well as individual associated risk and prognostic factors. A diagnostic approach is needed that encompasses a wide range of chromosomal abnormalities. Advanced gene expression studies are required to detect mutations responsible for frontline drug resistance. Such advanced techniques hold particular promise for creating personalized treatments at select treatment centers.

For CAR-T therapy, pharmaceutical companies are engineering T cells that can recognize tumor-specific antigens on cancer cells. Significant efforts are needed to continue identifying target biomarkers on cancer cells for monitoring DLBCL outcomes after treatment. The ideal targets for CAR-T cells are tumor-specific antigens that are homogeneously expressed on the surface of malignant cells, and which play critical roles in tumorigenesis. CAR-T cells may remain inside the body long after the infusion is complete. They protect against recurrence of cancer so the therapy often leads to long-term remissions. Researchers and doctors call it a “living drug” whose action is mediated by permanently altered cells that persist and multiply *in vivo*, continuing to fight the disease. The data from clinical trials released by Novartis and Kite Pharmaceuticals are promising, as 43% and 36%, respectively, of patients, had a complete response after six months of CAR-T therapy. FDA has been approved CAR-T therapy (Clinical trials of chimeric genetically modified T cells for CD19 by Kite Pharma) for diffuse large B-cell lymphoma (DLBCL), high-grade B-cell lymphoma, and DLBCL arising from follicular lymphoma (transformed follicular lymphoma, or TFL), not otherwise specified. However, a risk assessment and mitigation strategy is approved, and risks include syndrome of cytokine release (CRS), viral reactivation, prolonged cytopenias, and neurological toxicity.

A growing list of novel agents targeting specific pathways are currently under clinical investigation and these may improve the ORR, PFS, and OS for relapsed/refractory DLBCL patients. Fully developing an approach to prioritize traditional and novel therapeutic agents that affect key driver pathways, and combining those drugs to create new, multipronged treatment modalities that will provide a standard for individual risk-adapted therapy is the future for DLBCL treatment and cancer therapy generally.

## SUPPLEMENTARY MATERIALS





## Abbreviations

DLBCL: Diffuse large B-cell lymphoma
NHL: Non-Hodgkin lymphoma
DLBCL-NOS: Diffuse Large B-cell lymphoma not otherwise specified
GEP: Gene expression profiling
GCB-type DLBCL: Germinal center B-cell type Diffuse Large B-cell lymphoma
ABC-type DLBCL: Activated B-cell type Diffuse Large B-cell lymphoma
R-CHOP: Rituximab, cyclophosphamide, doxorubicin, vincristine, and prednisone
CARD11: caspase recruitment domain 11
DHL: Double-hit lymphoma
CAR-T: Chimeric antigen receptor T cells
SEER: Surveillance, Epidemiology, and End Results Program
CNS: Central Nervous System
LN: Lymph Node
SLL: Small lymphocytic leukemia
IVD: *In Vitro* Diagnostic
IHC: immunohistochemistry
FISH: Fluorescent *in situ* hybridization
Array CGH: Array comparative genomic hybridization
NGS: Next-generation sequencing
NCCN: National Comprehensive Cancer Network
ASR: Analyze specific reagent
OS: Overall survival
PFS: Progression-free survival. SKY: Spectral karyotyping
FISH: Fluorescence *in situ* hybridization
iFISH: interphase FISH
MCL: Mantle cell lymphoma
PET: Positron emission tomography
CLL: Chronic lymphocytic leukemia
SNP: Small Nucleotide Polymorphism
FFPE: Formalin-fixed, paraffin-embedded
qNPA: Quantitative nuclease protection assay
WB: Western blotting
ELISA: Enzyme-Linked Immunosorbent Assay
PCR: Polymerase Chain Reaction
GC: Germinal center
BAFF: B-cell–activating factor
TLRs: Toll-like receptors
miRNAs: MicroRNAs
CHOP: Cyclophosphamide, doxorubicin, vincristine, and prednisone
EFS: Event-free survival
ORR: Overall response rate
PR: Partial response
RT: Radiotherapy
DHAP: Dexamethasone, Cisplatin, Cytarabine
GDP: Gemcitabine, Dexamethasone, Cisplatin
HD-ASCT: high-dose chemotherapy followed by autologous stem cell transplant
ESHAP: Etoposide, methylprednisolone cytarabine, cisplatin
ICE: Ifosfamide, carboplatin, etoposide
MINE: Mesna, ifosfamide, mitoxantrone, etoposide
CEPP: Cyclophosphamide, etoposide, prednisone, procarbazine
CEOP: Cyclophosphamide, etoposide, vincristine, prednisone
HNSCC: Head and neck squamous cell cancer
PD- 1: Programmed cell death 1
CTLA: 4: Cytotoxic T-lymphocyte-associated antigen 4
Ipi-rt: Ipilimumab-radiotherapy
CR: complete response
TME: Tumor microenvironment. BTK: Bruton’s tyrosine kinase
STK: Spleen tyrosine kinase
EZH2: Enhancer of zeste hormone 2
PI3K: phosphoinositide-3-kinase
mTOR: mammalian Target of rapamycin. JAK: Janus Kinase
BCL: B-cell lymphoma
RVR: Everolimus in combination with Bortezomib and Rituximab
CBM: CARD11-BCL10-MALT1
STAT: Signal transducer and activator of transcription
PRC2: Polycomb-repressive complex 2
