# Supplementary material for: Biomarkers and novel therapeutic approaches for diffuse large B-cell lymphoma in the era of precision medicine
Source: Oncotarget. 2020 Nov 3;11(44):4045–73. doi: 10.18632/oncotarget.27785 (PMC7646825; doi:10.18632/oncotarget.27785)
Supplement: Supplementary file 1 [file oncotarget-11-4045-s001.pdf]

Biomarkers and novel therapeutic approaches for diffuse large B-cell lymphoma in the era of precision medicine

SUPPLEMENTARY MATERIALS

Supplementary Table 1: List of the diagnostic assays and antibodies that are utilized in the detection of DLBCL and/or its biomarkers. See Supplementary Tables File

Supplementary Table 2: List of key DLBCL biomarkers their normal functions and their role in diagnosis and prognosis for DLBCL along with their functional drug interactions of DLBCL. See Supplementary Tables File

Supplementary Table 3: Table describing the details of published and completed clinical trials on untreated and relapse/refractory form of DLBCL where therapeutic agents were tested as single agents or in combination. See Supplementary Tables File

Supplementary Table 4: Table describing the status of ongoing clinical trials registered with NIH at clinicaltrials.gov for DLBCL. See Supplementary Tables File

Supplementary Table 5: Describes the pharmacological details of drugs currently in use for treatment of DLBCL. See Supplementary Tables File

Supplementary Table 6: Table describing the pharmacological details of drugs currently under investigation for treatment of DLBCL. See Supplementary Tables File

SUPPLEMENTARY REFERENCES

1. Biogenex. Anti-BCl-2 [EP36] product webpage. 2016.
2. Biogenex. Anti-BCl6 [LN22] Datasheet. Doc. No. 932-708M-EN. 2014; Rev. A.
3. Hunt K, Reichard KK. Diffuse large B-cell lymphoma. Arch Pathol Lab Med. 2008; 132:118–24. [PubMed]
4. Biogenex. Anti-CD10 [56C6] Datasheet. Doc. No. 932-451M-EN. 2011. Rev. C.
5. Biogenex. Anti-human ki-67 product webpage. 2016.
6. Li ZM, Huang JJ, Xia Y, Zhu YJ, Zhao W, Wei WX, Jiang WQ, Lin TY, Huang HQ, Guan ZZ. High Ki-67 expression in diffuse large B-cell lymphoma patients with non-germinal center subtype indicates limited survival benefit from R-CHOP therapy. Eur J Haematol. 2012; 88:510–517. https://doi.org/10.1111/j.1600-0609.2012.01778.x. [PubMed]
7. Biogenex. Anti-Mum/IRF4 product webpage. 2016.
8. Hassan U, Ishtiaq S, Hussain M. Antibodies and Immediate Clinical Response in Patients of Diffuse Large B-Cell Lymphomas after Six Cycles of Chemotherapy. J Coll Physicians Surg Pak. 2014; 24:722–727. https://doi.org/10.2014/JCPSP.722727. [PubMed]
9. Biogenex. Anti-CD20 (B cell) [L-26] Data sheet. Doc. No. 932-238M-En 2011. Rev. B.
10. Johnson NA, Boyle M, Bashashati A, Leach S, Brooks-Wilson A, Sehn LH, Chhanabhai M, Brinkman RR, Connors JM, Weng AP, Gascoyne RD. Diffuse large B-cell lymphoma: reduced CD20 expression is associated with an inferior survival. Blood. 2009; 113:3773–3780. https://doi.org/10.1182/blood-2008-09-177469. [PubMed]
11. Sigma-Aldrich Co. LLC. CD5 (EP77) Rabbit Monoclonal Antibody webpage. 2016.
12. Miyazaki K, Yamaguchi M, Imai H, Kobayashi K, Tamaru S, Kobayashi T, Shiku H, Katayama N. Gene expression profiling of diffuse large B-Cell lymphomas supervised by CD5 expression. Int J Hematol. 2015; 102:188–194. https://doi.org/10.1007/s12185-015-1812-2. [PubMed]
13. Diagnostic Biosystems. CD138 (EPI201) Rabbit Monoclonal Antibody Datasheet. Doc. Num. DS-0050-A. 2014.
14. Abd El-Maqsoud NM, Gayyed MF. Prognostic value of CD10, BCL-6, MUM-1, and CD138 in diffuse large B-cell lymphoma. Egyptian Journal of Pathology. 2015; 35:95–104. https://doi.org/10.1097/01.XEJ.0000465876.33342.db.
15. Diagnostic Biosystems. CD79A/B (RP125) Polyclonal Rabbit Anti-Human Datasheet. 2016.
16. Diagnostic Biosystems. CD3 (SP7) Rabbit Monoclonal Antibody Datasheet. Doc. Num. DS-0070-A. 2014.
17. Diagnostic Biosystems. CD45 (EP68) Rabbit Monoclonal Antibody Datasheet. Doc. Num. DS-0088-A. 2014.
18. Diagnostic Biosystems. CD30 (EPI54) Rabbit Monoclonal Antibody Datasheet. Doc. Num. DS-0075-B. 2014.
19. Hu S, Xu-Monette ZY, Balasubramanyam A, Manyam GC, Visco C, Tzankov A, Liu W, Miranda RN, Zhang L, Montes-Moreno S, Dybkær K, Chiu A, Orazi A, et al. CD30 expression defines a novel subgroup of diffuse large B-cell lymphoma with favorable prognosis and distinct gene expression signature: a report from the International DLBCL Rituximab-CHOP Consortium Program Study. Blood. 2013; 121:2715–2724. https://doi.org/10.1182/blood-2012-10-461848. [PubMed]
20. CytoTest. BCL2 Break Apart FISH Probe Kit Datasheet. MK-DS-PAC206-EN. 2015; 03:01.
21. CytoTest. BCL6 Break Apart FISH Probe Kit Datasheet. MK-DS-PAC207-EN. 2015; 03:01.
22. Knight Diagnostic Laboratories. Diffuse Large B-Cell Lymphoma (DLBCL) FISH Panel product webpage. 2016.
23. HTG Molecular. HTG EdgeSeq Lymphoma Panel sales sheet. 2016. Rev. 3.
24. CytoTest. FOXO1 FISH Probe Kit Datasheet. MK-DS-PAC045-EN. 2015; 03:01.
25. CytoTest. JAK2 FISH Probe Kit Datasheet. MK-DS-PAC174-EN. 2015; 11:01.
26. CytoTest. MDM2/CCP12 FISH Probe Kit Datasheet. MK-DS-PAC019-EN. 2015; 03:01.
27. CytoTest. PTEN/CCP10 FISH Probe Kit Datasheet. MK-DS-COR101-EN. 2013; 03:01.
28. CytoTest. RB1/LAMP1 FISH Probe Kit Datasheet. MK-DS-PAC354-EN. 2015; 03:01.
29. CytoTest. TP53/CD37 FISH Probe Kit Datasheet. MK-DS-PAC157-EN. 2015; 08:01.
30. CytoTest. MYC FISH Probe Kit Datasheet. MK-DS-PAC208-EN. 2015; 03:01.
31. Abbott Molecular. Vysis LSI BCL2 Dual Color Break Apart Rearrangement Probe product description webpage. 2016.
32. Abbott Molecular. Vysis LSI IGH/BCL2 Dual Color, Dual Fusion Translocation Probe product description webpage. 2016.
33. Abbott Molecular. Vysis LSI MYC Dual Color Break Apart Rearrangement probe product description webpage. 2016.
34. Abbott Molecular. Vysis MDM2/CEP 12 FISH Probe Kit product description webpage. 2016.
35. ARUP Laboratories. Lymphoma (Aggressive) Panel by FISH additional technical information sheet. 2015.
36. BCL6. (3q27) Gene Rearrangement by FISH | ARUP Lab Test Directory. Available from: https://ltd.aruplab.com/Tests/Pub/3001311.
37. ARUP Laboratories. IGH-MYC Fusion by FISH additional technical information sheet. 2016.
38. ARUP Laboratories. MYC (8q24) Gene Rearrangement by FISH additional technical information sheet. 2016.
39. Lymphoma Phenotyping by Flow Cytometry additional technical information sheet. 2015.
40. HTG Molecular. HTG EdgeSeq DLBCL Cell of Origin Assay Brochure. 2016. Rev. 1.
41. Shen J, Xu L, Zhao Q. Perifosine and ABT-737 synergistically inhibit lung cancer cells *in vitro* and *in vivo*. Biochem Biophys Res Commun. 2016; 473:1170–1176. https://doi.org/10.1016/j.bbrc.2016.04.035. [PubMed]

42. Yi S, Zou D, Li C, Zhong S, Chen W, Li Z, Xiong W, Liu W, Liu E, Cui R, Ru K, Zhang P, Xu Y, et al. High incidence of MYC and BCL2 abnormalities in mantle cell lymphoma, although only MYC abnormality predicts poor survival. Oncotarget. 2015; 6:42362–42371. https://doi.org/10.18632/oncotarget.5705. [PubMed]
43. Gualco G, Weiss LM, Harrington WJ, Bacchi CE. BCL6, MUM1, and CD10 expression in mantle cell lymphoma. Appl Immunohistochem Mol Morphol. 2010; 18:103–108. https://doi.org/10.1097/PAI.0b013e3181bb9edf. [PubMed]
44. Liu Z, Davidson A. BAFF inhibition: a new class of drugs for the treatment of autoimmunity. Exp Cell Res. 2011; 317:1270–1277. https://doi.org/10.1016/j.yexcr.2011.02.005. [PubMed]
45. Burger JA. Bruton's Tyrosinase Kinase (BTK) inhibitors in clinical trials. Curr Hematol Malig Rep. 2014; 9:44–49. https://doi.org/10.1007/s11899-013-0188-8. [PubMed]
46. Wang ML, Rule S, Martin P, Goy A, Auer R, Kahl BS, Jurczak W, Advani RH, Romaguera JE, Williams ME, Barrientos JC, Chmielowska E, Radford J, et al. Targeting BTK with ibrutinib in relapsed or refractory mantle cell lymphoma. N Engl J Med. 2013; 369:507–516. https://doi.org/10.1056/NEJMoa1306220. [PubMed]
47. Camicia R, Winkler HC, Hassa PO. Novel drug targets for personalized precision medicine in relapsed/refractory diffuse large B-cell lymphoma: a comprehensive review. Molecular Cancer. 2015; 14. https://doi.org/10.1186/s12943-015-0474-2.
48. Chipumuro E, Marco E, Christensen CL, Kwiatkowski N, Zhang T, Hatheway CM, Abraham BJ, Sharma B, Yeung C, Altabef A, Perez-Atayde A, Wong KK, et al. CDK7 inhibition suppresses super-enhancer-linked oncogenic transcription in MYCN-driven cancer. Cell. 2014; 159:1126–1139. https://doi.org/10.1016/j.cell.2014.10.024. [PubMed]
49. Pasqualucci L, Dominguez-Sola D, Chiarenza A, Fabbri G, Grunn A, Trifonov V, Kasper LH, Lerach S, Tang H, Ma J, Rossi D, Chadburn A, Murty VV, et al. Inactivating mutations of acetyltransferase genes in B-cell lymphoma. Nature. 2011; 471:189–95. https://doi.org/10.1038/nature09730.
50. Benada J, Macurek L. Targeting the checkpoint to kill cancer cells. Biomolecules. 2015; 5:1912–1937. https://doi.org/10.3390/biom5031912. [PubMed]
51. Yamaguchi M, Seto M, Okamoto M, Ichinohasama R, Nakamura N, Yoshino T, Suzumiya J, Murase T, Miura I, Akasaka T, Tamaru J, Suzuki R, Kagami Y, et al. De novo CD5+ diffuse large B-cell lymphoma: a clinicopathologic study of 109 patients. Blood. 2002; 99:815–821. https://doi.org/10.1182/blood.V99.3.815. [PubMed]
52. Yamaguchi M, Ohno T, Oka K, Taniguchi M, Ito M, Kita K, Shiku H. De novo CD5-positive diffuse large B-cell lymphoma: clinical characteristics and therapeutic outcome. Br J Haematol. 1999; 105:1133–1139. https://doi.org/10.1046/j.1365-2141.1999.01513.x. [PubMed]
53. Xu Y, McKenna RW, Molberg KH. Clinical relevance of CD10 expression in de novo large B-cell lymphoma. Mod Pathol. 2000; 13:166A.
54. Fang JM, Finn WG, Hussong JW, Goolsby CL, Cubbon AR, Variakojis D. CD10 antigen expression correlates with the t(14;18)(q32;q21) major breakpoint region in diffuse large B-cell lymphoma. Mod Pathol. 1999; 12:295–300. [PubMed]
55. Friedberg JW, Fisher RI. Iodine-131 tositumomab (Bexxar): radioimmunoconjugate therapy for indolent and transformed B-cell non-Hodgkin's lymphoma. Expert Rev Anticancer Ther. 2004; 4:18–26. https://doi.org/10.1586/14737140.4.1.18. [PubMed]
56. Ely SA, Knowles DM. Expression of CD56/neural cell adhesion molecule correlates with the presence of lytic bone lesions in multiple myeloma and distinguishes myeloma from monoclonal gammopathy of undetermined significance and lymphomas with plasmacytoid differentiation. Am J Pathol. 2002; 160:1293–1299. https://doi.org/10.1016/S0002-9440(10)62556-4. [PubMed]
57. Challa-Malladi M, Lieu YK, Califano O, Holmes AB, Bhagat G, Murty VV, Dominguez-Sola D, Pasqualucci L, Dalla-Favera R. Combined genetic inactivation of β2-Microglobulin and CD58 reveals frequent escape from immune recognition in diffuse large B cell lymphoma. Cancer Cell. 2011; 20:728–740. https://doi.org/10.1016/j.ccr.2011.11.006. [PubMed]
58. Naylor TL, Tang H, Ratsch BA, Enns A, Loo A, Chen L, Lenz P, Waters NJ, Schuler W, Dörken B, Yao Y-M, Warmuth M, Lenz G, et al. Protein kinase C inhibitor sotrastaurin selectively inhibits the growth of CD79 mutant diffuse large B-cell lymphomas. Cancer Res. 2011; 71:2643–53. https://doi.org/10.1158/0008-5472.CAN-10-2525.
59. Oh YH, Park CK. Prognostic evaluation of nodal diffuse large B cell lymphoma by immunohistochemical profiles with emphasis on CD138 expression as a poor prognostic factor. J Korean Med Sci. 2006; 213:397–405. https://doi.org/10.3346/jkms.2006.21.3.397. [PubMed]
60. CD274 Gene - GeneCards | PD1L1 Protein | PD1L1 Antibody. Available from: https://www.genecards.org/cgibin/carddisp.pl?gene=CD274&keywords=CD274.
61. EZH2 Gene - GeneCards | EZH2 Protein | EZH2 Antibody. Available from: https://www.genecards.org/cgi-bin/carddisp.pl?gene=EZH2&keywords=EZH2.
62. Baas T. OutFOXing tumors. SciBX; 2012:5.
63. Barrans SL, Fenton JA, Banham A, Owen RG, Jack AS. Strong expression of FOXP1 identifies a distinct subset of diffuse large B-cell lymphoma (DLBCL) patients with poor outcome. Blood. 2004; 104:2933–2935. https://doi.org/10.1182/blood-2004-03-1209. [PubMed]
64. HLA-DRA Gene - GeneCards | DRA Protein | DRA Antibody. Available from: https://www.genecards.org/cgi-bin/carddisp.pl?gene=HLA-DRA&keywords=MHC,Class,II,antigen,DRA.
65. HLA-DRB1 Gene - GeneCards | DRB Protein | DRB Antibody. Available from: https://www.genecards.org/cgi-bin/carddisp.pl?gene=HLA-DRB1&keywords=MHC,Class,II,antigen,DRA.
66. GeneCards - Human Genes | Gene Database | Gene Search. Available from: https://www.genecards.org.

67. Rodriguez-Burford C, Barnes MN, Oelschlager DK, Myers RB, Talley LI, Partridge EE, Grizzle WE. Effects of nonsteroidal anti-inflammatory agents (NSAIDs) on ovarian carcinoma cell lines: preclinical evaluation of NSAIDs as chemopreventive agents. Clin Cancer Res. 2002; 8:202–209. [PubMed]

68. Natkunam Y, Farinha P, Hsi ED, Hans CP, Tibshirani R, Sehn LH, Connors JM, Gratzinger D, Rosado M, Zhao S, Pohlman B, Wongchaowart N, Bast M, et al. LMO2 protein expression predicts survival in patients with diffuse large B-cell lymphoma treated with anthracycline-based chemotherapy with and without rituximab. J Clin Oncol. 2008; 263:447–454. https://doi.org/10.1200/JCO.2007.13.0690. [PubMed]

69. Xu-Monette ZY, Moller MB, Tzankov A, Montes-Moreno S, Hu W, Manyam GC, Kristensen L, Fan L, Visco C, Dybkaer K, Chiu A, Tam W, Zu Y, et al. MDM2 phenotypic and genotypic profiling, respective to TP53 genetic status, in diffuse large B-cell lymphoma patients treated with rituximab-CHOP immunochemotherapy: a report from the International DLBCL Rituximab-CHOP Consortium Program. Blood. 2013; 122:2630–40. https://doi.org/10.1182/blood-2012-12-473702.

70. Lim EL, Trinh DL, Scott DW, Chu A, Krzywinski M, Zhao Y, Robertson AG, Mungall AJ, Schein J, Boyle M, Mottok A, Ennishi D, Johnson NA, et al. Comprehensive miRNA sequence analysis reveals survival differences in diffuse large B-cell lymphoma patients. Genome Biology. 2015; 16:18. https://doi.org/10.1186/s13059-014-0568-y.

71. Mazan-Mamczarz K, Gartenhaus RB. Role of microRNA deregulation in the pathogenesis of diffuse large B-cell lymphoma (DLBCL). Leukemia Research. 2013; 37:1420–8. https://doi.org/10.1016/j.leukres.2013.08.020.

72. Liu P, Cheng H, Roberts TM, Zhao JJ. Targeting the phosphoinositide-3-kinase (PI3K) pathway in cancer. Nature Reviews. 2009; 8:627–644.

73. Davies C, Hogarth LA, Mackenzie KL, Hall AG, Lock RB. P21 (WAF1) modulates drug-induced apoptosis and cell cycle arrest in B cell precursor acute lymphoblastic leukemia. Cell Cycle. 2015; 1422:3602–3612. https://doi.org/10.1080/15384101.2015.1100774. [PubMed]

74. Youn J, Lee K, Lee C, Gabrilovich D. Loss of Rb1 by epigenetic modification regulates expansion of MDSC in cancer. J Immunother Cancer. 2014; 2:241. https://doi.org/10.1186/2051-1426-2-S3-P241.

75. Yang Y, Shaffer AL, Emre NC, Ceribelli M, Zhang M, Wright G, Xiao W, Powell J, Platig J, Kohlhammer H, Young RM, Zhao H, Yang Y, et al. Exploiting synthetic lethality for the therapy of ABC diffuse large B cell lymphoma. Cancer Cell. 2012; 21:723–737. https://doi.org/10.1016/j.ccr.2012.05.024. [PubMed]

76. Park J, Ahn KS, Bae EK, Kim BK, Lee YY, Yoon SS. Blockage of interleukin-6 signaling with 6-amino-4-quinazoline synergistically induces the inhibitory effect of bortezomib in human U266 cells. Anticancer Drugs. 2008; 198:777–782. https://doi.org/10.1097/CAD.0b013e32830c236a. [PubMed]

77. Yue P, Turkson J. Targeting STAT3 in cancer: how successful are we? Expert Opin Investig Drugs. 2009; 18:45–56. https://doi.org/10.1517/13543780802565791. [PubMed]

78. Lu K, Chen N, Zhou XX, Ge XL, Feng LL, Li PP, Li XY, Geng LY, Wang X. The STAT3 inhibitor WP1066 synergizes with vorinostat to induce apoptosis of mantle cell lymphoma cells. Biochem Biophys Res Commun. 2015; 464:292–298. https://doi.org/10.1016/j.bbrc.2015.06.145. [PubMed]

79. Zhou W, Zhang J, Goleniewska K, Dulek DE, Toki S, Newcomb DC, Cephus JY, Collin RD, Wu P, Boothby MR, Peebles RS. Prostaglandin I2 suppresses proinflammatory chemokine expression, CD4 T cell activation, and STAT6 independent allergic lung inflammation. J Immunol. 2016; 197:1577–1586. https://doi.org/10.4049/jimmunol.1501063. [PubMed]

80. Halldorsdottir AM, Lundin A, Murray F, Mansouri L, Knuutila S, Sundstrom C, Laurell A, Ehrencrona H, Sander B, Rosenquist R. Impact of Tp53 mutation and 17p deletion in mantle cell lymphoma. Leukemia. 2011; 25:1904–1908. https://doi.org/10.1038/leu.2011.162. [PubMed]

81. Nordstrom L, Sernbo S, Eden P, Gronbaek K, Kolstad A, Raty R, Karjalainen ML, Geisler C, Ralfkiaer E, Sundstrom C, Laurell A, Delabie J, Ehinger M, et al. SOX11 and Tp53 add prognostic information to MIPI in a homogenously treated cohort of mantle cell lymphoma-a Nordic lymphoma group study. Br J Haematol. 2014; 166:98–108. https://doi.org/10.1111/bjh.12854. [PubMed]

82. Wei Y, Melas PA, Wegener G, Mathe AA, Lavebratt C. Antidepressant-like effect of sodium butyrate is associated with an increase in TET1 and in 5-hydroxymethylation levels in the Bdnf gene. Int J Neuropsychopharmacol. 2014; 18:pyu032. https://doi.org/10.1093/ijnp/pyu032. [PubMed]

83. Verstrepen L, Carpentier I, Verhelst K, Beyaert R. ABINs: A20 binding inhibitors of NF-kappa B and apoptosis signaling. Biochem Pharmacol. 2009; 78:105–114. https://doi.org/10.1016/j.bcp.2009.02.009. [PubMed]

84. Edwards SK, Han Y, Liu Y, Kreider BZ, Liu Y, Grewal S, Desai A, Baron J, Moore CR, Luo C, Xie P. Signaling mechanisms of bortezomib in TRAF3-deficient mouse B lymphoma and human multiple myeloma cells. Leuk Res. 2016; 41:85–95. https://doi.org/10.1016/j.leukres.2015.12.005. [PubMed]

85. Rasmussen KD, Jia G, Johansen JV, Pedersen MT, Rapin N, Bagger FO, Porse BT, Bernard OA, Christensen J, Helin K. Loss of TET2 in hematopoietic cells leads to DNA hypermethylation of active enhancers and induction of leukemogenesis. Genes Dev. 2015; 29:910–922. https://doi.org/10.1101/gad.260174.115. [PubMed]

86. Solary E, Bernard OA, Tefferi A, Fuks F, Vainchenker W. The Ten-Eleven Translocation-2 (TET2) gene in hematopoiesis and hematopoietic diseases. Leukemia. 2014; 28:485–496. https://doi.org/10.1038/leu.2013.337. [PubMed]

87. Delhommeau F, Dupont S, Della Valle V, James C, Trannoy S, Massé A, Kosmider O, Le Couedic JP, Robert F, Alberdi A, Lécluse Y, Plo I, Dreyfus FJ, et al. Mutation in TET2

in myeloid cancers. N Engl J Med. 2009; 360:2289–2301. https://doi.org/10.1056/NEJMoa0810069. [PubMed]

88. Asmar F, Punj V, Christensen J, Pedersen MT, Pedersen A, Nielsen AB, Hother C, Ralfkiaer U, Brown P, Ralfkiaer E, Helin K, Grønbæk K. Genome-wide profiling identifies a DNA methylation signature that associates with TET2 mutations in diffuse large B-cell lymphoma. Haematologica. 2013; 98:1912–1920. https://doi.org/10.3324/haematol.2013.088740. [PubMed]

89. Sehn LH, Martelli M, Trněný M, Liu W, Bolen CR, Knapp A, Sahin D, Sellam G, Vitolo U. A randomized, open-label, Phase III study of obinutuzumab or rituximab plus CHOP in patients with previously untreated diffuse large B-Cell lymphoma: final analysis of GOYA. J Hematol Oncol. 2020; 13:71. https://doi.org/10.1186/s13045-020-00900-7.

90. Vacirca JL, Acs PI, Tabbara IA, Rosen PJ, Lee P, Lynam E. Bendamustine combined with rituximab for patients with relapsed or refractory diffuse large B cell lymphoma. Ann Hematol. 2014; 93:403–409. https://doi.org/10.1007/s00277-013-1879-x. [PubMed]

91. Micallef IN, Kahl BS, Maurer MK, Dogan A, Ansell SM, Colgan JP, Geyer S, Inwards DJ, White WL, Habermann TM. A pilot study of epratuzumab and rituximab in combination with cyclophosphamide, doxorubicin, vincristine, and prednisone chemotherapy in patients with previously untreated, diffuse large B- cell lymphoma. Cancer. 2006; 107:2826–2832. https://doi.org/10.1002/cncr.22342. [PubMed]

92. Feugier P, Van Hoof A, Sebban C, Solal-Celigny P, Bouabdallah R, Fermé C, Christian B, Lepage E, Tilly H, Morschhauser F, Gaulard P, Salles G, Bosly A, et al. Long-term results of the R-CHOP study in the treatment of elderly patients with diffuse large B-cell lymphoma: a study by the Groupe d'Etude des Lymphomes de l'Adulte. J Clin Oncol. 2005; 23:4117–4126. https://doi.org/10.1200/JCO.2005.09.131. [PubMed]

93. Levine AM, Noy A, Lee JY, Tam W, Ramos JC, Henry DH, Parekh S, Reid EG, Mitsuyasu R, Cooley T, Dezube BJ, Ratner L, Cesarman E, et al. Pegylated liposomal doxorubicin, rituximab, cyclophosphamide, vincristine, and prednisone in AIDS-related lymphoma: AIDS Malignancy Consortium Study 047. J Clin Oncol. 2013; 1:58–64. https://doi.org/10.1200/JCO.2012.42.4648. [PubMed]

94. Stopeck AT, Unger JM, Rimsza LM, LeBlanc M, Farnsworth B, Iannone M, Glenn MJ, Fisher RI, Miller TP. A phase 2 trial of standard-dose cyclophosphamide, doxorubicin, vincristine, prednisone (CHOP) and rituximab plus bevacizumab for patients with newly diagnosed diffuse large B-cell non-Hodgkin lymphoma: SWOG 0515. Blood. 2012; 120:1210–1217. https://doi.org/10.1182/blood-2012-04-423079. [PubMed]

95. Sparano JA, Lee JY, Kaplan LD, Levine AM, Ramos JC, Ambinder RF, Wachsman W, Aboulafia D, Noy A, Henry DH, Von Roenn J, Dezube BJ, Remick SC, et al. Rituximab plus concurrent infusional EPOCH chemotherapy is highly effective in HIV-associated B-cell non-Hodgkin lymphoma. Blood. 2010; 115:3008–3016. https://doi.org/10.1182/blood-2009-08-231613. [PubMed]

96. Riihijärvi S, Nurmi H, Holte H, Björkholm M, Fluge O, Pedersen LM, Rydström K, Jerkeman M, Eriksson M, Leppä S. High serum vascular endothelial growth factor level is an adverse prognostic factor for high-risk diffuse large B-cell lymphoma patients treated with dose-dense chemoimmunotherapy. Eur J Haematol. 2012; 89:395–402. https://doi.org/10.1111/ejh.12005. [PubMed]

97. Fields PA, Townsend W, Webb A, Counsell N, Pocock C, Smith P, Jack A, El-Mehidi N, Johnson PW, Radford J, Linch DC, Cunnningham D. *De novo* treatment of diffuse large B-cell lymphoma with rituximab, cyclophosphamide, vincristine, gemcitabine, and prednisolone in patients with cardiac comorbidity: a United Kingdom National Cancer Research Institute trial. J Clin Oncol. 2014; 32:282–287. https://doi.org/10.1200/JCO.2013.49.7586. [PubMed]

98. Mehra R, Seiwert TY, Mahipal A, Weiss J, Berger R, Eder JP, Burtness B, Tahara M, Keam B, Le DT, Muro K, Geva R, Chung HC, et al. Efficacy and safety of pembrolizumab in recurrent/metastatic head and neck squamous cell carcinoma (R/M HNSCC): Pooled analyses after long-term follow-up in KEYNOTE-012. J Clin Oncol. 2016; 34:6012–6012. https://doi.org/10.1200/JCO.2016.34.15_suppl.6012.

99. Kim JE, Yoon DH, Jang G, Lee DH, Kim S, Park CS. A phase I/II study of bortezomib plus CHOP every 2 weeks (CHOP-14) in patients with advanced-stage diffuse large B-cell lymphomas. Korean J Hematol. 2012; 47:53–59. https://doi.org/10.5045/kjh.2012.47.1.53. [PubMed]

100. Merli F, Luminari S, Rossi G, Mammi C, Marcheselli L, Tucci A, Ilariucci F, Chiappella A, Musso M, Di Rocco A, Stelitano C, Alvarez I, Baldini L, et al. Cyclophosphamide, doxorubicin, vincristine, prednisone and rituximab versus epirubicin, cyclophosphamide, vinblastine, prednisone and rituximab for the initial treatment of elderly "fit" patients with diffuse large B-cell lymphoma: results from the ANZINTER3 trial of the Intergruppo Italiano Linfomi. Leuk Lymphoma. 2012; 53:581–588. https://doi.org/10.3109/10428194.2011.621565. [PubMed]

101. Byrd JC, Peterson BL, Gabrilove J, Odenike OM, Grever MR, Rai K, Larson RA. Cancer and Leukemia Group B. Treatment of relapsed chronic lymphocytic leukemia by 72-hour continuous infusion or 1-hour bolus infusion of flavopiridol: results from Cancer and Leukemia Group B study 19805. Clin Cancer Res. 2005; 11:4176–4181. https://doi.org/10.1158/1078-0432.CCR-04-2276. [PubMed]

102. Neelapu SS, Locke FL, Bartlett NL, Lekakis LJ, Miklos DB, Jacobson CA, Braunschweig I, Oluwole OO, Siddiqi T, Lin Y, Timmerman JM, Stiff PJ, Friedberg JW, et al. Axicabtagene Ciloleucel CAR T-Cell Therapy in Refractory Large B-Cell Lymphoma. New England Journal of Medicine. 2017; 377:2531–44. https://doi.org/10.1056/NEJMoa1707447.

103. Crump M, Neelapu SS, Farooq U, Van Den Neste E, Kuruvilla J, Westin J, Link BK, Hay A, Cerhan JR, Zhu L, Boussetta S, Feng L, Maurer MJ, et al. Outcomes in refractory diffuse large B-cell lymphoma: results from the

international SCHOLAR-1 study. Blood. 2017; 130:1800–8. https://doi.org/10.1182/blood-2017-03-769620.

104. Wilson WH, Young RM, Schmitz R, Yang Y, Pittaluga S, Wright G, Lih CJ, Williams PM, Shaffer AL, Gerecitano J, de Vos S, Goy A, Kenkre VP, et al. Targeting B cell receptor signaling with ibrutinib in diffuse large B cell lymphoma. Nat Med. 2015; 21:922–926. https://doi.org/10.1038/nm.3884. [PubMed]

105. Oki Y, Fanale M, Romaguera J, Fayad L, Fowler N, Copeland A, Samaniego F, Kwak LW, Neelapu S, Wang M, Feng L, Younes A. Phase II study of an AKT inhibitor MK2206 in patients with relapsed or refractory lymphoma. Br J Haematol. 2015; 171:463–470. https://doi.org/10.1111/bjh.13603. [PubMed]

106. Jacobsen ED, Sharman JP, Oki Y, Advani RH, Winter JN, Bello CM, Spitzer G, Palanca-Wessels MC, Kennedy DA, Levine P, Yang J, Bartlett NL. Brentuximab vedotin demonstrates objective responses in a phase 2 study of relapsed/refractory DLBCL with variable CD30 expression. Blood. 2015; 125:1394–1402. https://doi.org/10.1182/blood-2014-09-598763. [PubMed]

107. Coiffier B, Radford J, Bosly A, Martinelli G, Verhoef G, Barca G, Davies A, Decaudin D, Gallop-Evans E, Padmanabhan-Iyer S, Van Eygen K, Wu KL, Gupta IV, et al. 415 study investigators. A multicentre, phase II trial of ofatumumab monotherapy in relapsed/progressive diffuse large B-cell lymphoma. Br J Haematol. 2013; 163:334–342. https://doi.org/10.1111/bjh.12537. [PubMed]

108. Moschetta M, Reale A, Marasco C, Vacca A, Carratù MR. Therapeutic targeting of the mTOR-signalling pathway in cancer: benefits and limitations. Br J Pharmacol. 2014; 171:3801–13. https://doi.org/10.1111/bph.12749.

109. Smith SM, van Besien K, Karrison T, Dancey J, McLaughlin P, Younes A, Smith S, Stiff P, Lester E, Modi S, Doyle LA, Vokes EE, Pro B. Temsirolimus has activity in non-mantle cell non-Hodgkin lymphoma subtypes: The University of Chicago phase II consortium. J Clin Oncol. 2010; 28:4740–4746. https://doi.org/10.1200/JCO.2010.29.2813. [PubMed]

110. Buckstein R, Kuruvilla J, Chua N, Lee C, Macdonald DA, Al-Tourah AJ. Sunitinib in relapsed or refractory diffuse large B-cell lymphoma: a clinical and pharmacodynamic phase II multicenter study of the NCIC Clinical Trials Group. Leuk Lymphoma. 2011; 52:833–841. https://doi.org/10.3109/10428194.2011.555892. [PubMed]

111. Horwitz SM, Negrin RS, Blume KG, Breslin S, Stuart MJ, Stockerl-Goldstein KE, Johnston LJ, Wong RM, Shizuru JA, Horning SJ. Rituximab as adjuvant to high-dose therapy and autologous hematopoietic cell transplantation for aggressive non-Hodgkin lymphoma. Blood. 2004; 103:777–783. https://doi.org/10.1182/blood-2003-04-1257. [PubMed]

112. Pro B, Leber B, Smith M, Fayad L, Romaguera J, Hagemeister F. Phase II multicenter study of oblimersen sodium, a Bcl-2 antisense oligonucleotide, in combination with rituximab in patients with recurrent B-cell non-Hodgkin lymphoma. Br J Haematol. 2008; 143:355–360. https://doi.org/10.1111/j.1365-2141.2008.07353.x. [PubMed]

113. Study of SyB L-0501 in Combination With Rituximab to Treat Relapsed/Refractory Diffuse Large B-Cell Lymphoma - Study Results - https://clinicaltrials.gov/ . Available from: https://clinicaltrials.gov/ct2/show/results/NCT01118845.

114. Foran JM, Cunningham D, Coiffier B, Solal-Celigny P, Reyes F, Ghielmini M, Johnson PW, Gissebrecht C, Bradburn M, Matthews J, Lister TA. Treatment of mantle-cell lymphoma with Rituximab (chimeric monoclonal anti-CD20 antibody): analysis of factors associated with response. Ann Oncol. 2000; 11:117–121. https://doi.org/10.1093/annonc/11.suppl_1.S117. [PubMed]

115. Fraiser LH, Kanekal S, Kehrer JP. Cyclophosphamide toxicity. Characterising and avoiding the problem. Drugs. 1991; 42:781–795. https://doi.org/10.2165/00003495-199142050-00005. [PubMed]

116. Tewey KM, Rowe TC, Yang L, Halligan BD, Liu LF. Adriamycin-induced DNA damage mediated by mammalian DNA topoisomerase-II. Science. 1984; 226:466–468. https://doi.org/10.1126/science.6093249. [PubMed]

117. Shi Y, Zhou P, Han X, He X, Zhou S, Liu P, Yang J, Zhang C, Gui L, Qin Y, Yang S, Zhao L, Yao J, et al. Autologous peripheral blood stem cell mobilization following dose-adjusted cyclophosphamide, doxorubicin, vincristine, and prednisolone chemotherapy alone or in combination with rituximab in treating high-risk non-Hodgkin's lymphoma. Clin J Cancer. 2015; 34:522–30. https://doi.org/10.1186/s40880-015-0045-3. [PubMed]

118. Belgaumi AF, Al-Bakrah M, Al-Mahr M, Al-Jefri A, Al-Musa A, Saleh M, Salim MF, Osman M, Osman L, El-Solh H. Dexamethasone-associated toxicity during induction chemotherapy for childhood acute lymphoblastic leukemia is augmented by concurrent use of daunomycin. Cancer. 2013; 97:2898–2903. https://doi.org/10.1002/cncr.11390. [PubMed]

119. Polovich M, White JM, Kelleher LO. Chemotherapy and biotherapy guidelines and recommendations for practice, 2nd ed. Oncology Nursing Society. 2005.

120. American Society of Health-System Pharmacists. ASHP Guidelines on handling hazardous drugs. Am J Health Syst Pharm. 2006; 63:1172–1193. https://doi.org/10.2146/ajhp050529.

121. US Department of Labor Occupational Safety and Health Administration (OSHA). OSHA technical manual-controlling occupational exposure to hazardous drugs. 2004; 165.

122. NIH. Recommendations for the safe handling of parenteral antineoplastic drugs. NIH Publications. 1983:83–2621.

123. AMA Council on Scientific Affairs. Guidelines for handling parenteral antineoplastics. JAMA. 1985; 253:1590–1592. https://doi.org/10.1001/jama.1985.03350350084026.

124. National Study Commission on Cytotoxic Exposure. Recommendations for Handling Cytotoxic Agents. 1984.

125. Rolski S. Method of preparing vindesine sulfate. US Patent 4259242. 1981.

126. Kaczkowski CH. Vindesine. Gale Encyclopedia of Cancer. 2016.

127. Adams J, Palombella VJ, Sausville EA, Johnson J, Destree A, Lazarus DD, Maas J, Pien CS, Prakash S, Elliott PJ. Proteasome inhibitors: a novel class of potent and effective antitumor agents. Cancer Res. 1999; 59:2615–2622. [PubMed]

128. National Center for Biotechnology Information. 154447-36-6. Compound Summary for CID 3973. PubChem Compound Database. 2005.

129. Gharbi S, Zvelebil MJ, Shuttleworth SJ, Hancox T, Saghir N, Timms JF, Waterfield MD. Exploring the Specificity of the PI3K Family Inhibitor LY294002. Biochem J. 2007; 404:15–21. https://doi.org/10.1042/BJ20061489. [PubMed]

130. National Center for Biotechnology Information. CAL-101. Compound Summary for CID 11625818. PubChem Compound Database. 2006.

131. Raedler LA. Zydelig (Idelalisib): First-in-Class PI3 Kinase Inhibitor Approved for the Treatment of 3 Hematologic Malignancies. Am Health Drug Benefits. 2015; 8:157–162. [PubMed]

132. National Center for Biotechnology Information. ABT-737. Compound Summary for CID 11228183. PubChem Compound Database. 2006.

133. Konopleva M, Contractor R, Tsao T, Samudio I, Ruvolo PP, Kitada S, Deng X, Zhai D, Shi YX, Sneed T, Verhaegen M, Soengas M, Ruvolo VR, et al. Mechanisms of apoptosis sensitivity and resistance to the BH3 mimetic ABT-737 in acute myeloid leukemia. Cancer Cell. 2006; 10:375–388. https://doi.org/10.1016/j.ccr.2006.10.006. [PubMed]

134. National Center for Biotechnology Information. Navitoclax. Compound Summary for CID 24978538. 2008.

135. Wilson WH, O'Connor OA, Czuczman MS, LaCasce AS, Gerecitano JF, Leonard JP, Tulpule A, Dunleavy K, Xiong H, Chiu YL, Cui Y, Busman T, Elmore SW, et al. Safety, Pharmacokinetics, Pharmacodynamics, and Activity of Navitoclax, a Targeted High Affinity Inhibitor of BCL-2, in Lymphoid Malignancies. Lancet Oncol. 2010; 11:1149–1159. https://doi.org/10.1016/S1470-2045(10)70261-8. [PubMed]

136. Richardson PG, Eng C, Kolesar J, Hideshima T, Anderson KC. Perifosine, an oral, anti-cancer agent and inhibitor of the Akt pathway: mechanistic actions, pharmacodynamics, pharmacokinetics, and clinical activity. Expert Opin Drug Metab Toxicol. 2012; 8:623–633. https://doi.org/10.1517/17425255.2012.681376. [PubMed]

137. Ye XN, Zhou XP, Wei JY, Xu GX, Li Y, Mao LP, Huang J, Ren YL, Mei C, Wang JH, Lou YJ, Ma LY, Yu WJ, et al. Epigenetic priming with decitabine followed by low-dose idarubicin/cytarabine has an increased anti-leukemic effect compared to traditional chemotherapy in high-risk myeloid neoplasms. Leuk Lymphoma. 2016; 57:1311–1318. https://doi.org/10.3109/10428194.2015.1091931. [PubMed]

138. Kaplan MA, Granatek AP. Process for the preparation of microcrystalline cisplatin. U.S. Patent US4322391, issued March 30, 1982.

139. Dechant KL, Brogden RN, Pilkington T, Faulds D. Ifosfamide/mesna. A review of its antineoplastic activity, pharmacokinetic properties and therapeutic efficacy in cancer. Drugs. 1991; 42:428–467. https://doi.org/10.2165/00003495-199142030-00006. [PubMed]

140. Citterio G, Reni M, Ferreri AJ. Present and future treatment options for primary CNS lymphoma. Expert Opin Pharmacother. 2015; 16:2569–79. https://doi.org/10.1517/14656566.2015.1088828. [PubMed]

141. U.S Food and Drug Administration. Mitoxantrone Hydrochloride-healthcare professional sheet text version. 2015.

142. Pharmacia and Upjohn Company. Ellence full prescribing information text version. 2007.

143. Valdez BC, Andersson BS. Interstrand crosslink inducing agents in pretransplant conditioning therapy for hematologic malignancies. Environ Mol Mutagen. 2010; 51:659–668. [PubMed]

144. Loeber R, Michaelson E, Fang Q, Campbell C, Pegg AE, Tretyakova N. Cross-linking of the DNA repair protein Omicron6-alkylguanine DNA alkyltransferase to DNA in the presence of antitumor nitrogen mustards. Chem Res Toxicol. 2008; 21:787–795. https://doi.org/10.1021/tx7004508. [PubMed]

145. Weigel JA. Process for making gemcitabine hydrochloride. U.S. Patent US6001994. 1995.

146. Laurenti L, Innocenti I, Autore F, Sica S, Efremov DG. New developments in the management of chronic lymphocytic leukemia: role of ofatumumab. Onco Targets Ther. 2016; 9:421–429. https://doi.org/10.2147/OTT.S72845. [PubMed]

147. Ma LY, Su L. Application of Lenalidomide on Diffused Large B-cell Lymphoma: Salvage, Maintenance, and Induction Treatment. Chin Med J (Engl). 2018; 131:2510–2513. https://doi.org/10.4103/0366-6999.243567. [PubMed]

148. Miao Y, Medeiros LJ, Xu-Monette ZY, Li J, Young KH. Dysregulation of Cell Survival in Diffuse Large B Cell Lymphoma: Mechanisms and Therapeutic Targets. Front Oncol. 2019. https://doi.org/10.3389/fonc.2019.00107. [PubMed]

149. Siedlecki P, Martin D. Preparation of nitrogen mustard derivatives. U.S. Patent US20030162990. 2003.

150. Spivack SD. Drugs 5 years later: procarbazine. Ann Intern Med. 1974; 81:795–800. https://doi.org/10.7326/0003-4819-81-6-795. [PubMed]

151. Sigma Tau Pharmaceuticals, inc. Product Monograph-Procarbazine. 2008.

152. Wang M, Popplewell LL, Collins RH Jr, Winter JN, Goy A, Kaminski MS, Bartlett NL, Johnston PB, Lister J, Fanning SR, Tuscano JM, Beck JT, Kaya H, et al. Everolimus for patients with mantle cell lymphoma refractory to or intolerant of bortezomib: multicentre, single-arm, phase 2 study. Br J Haematol. 2014; 165:510–518. https://doi.org/10.1111/bjh.12780. [PubMed]

153. Lin TS, Andritsos AL, Jones JA, Fischer B, Heerema NA, Blum KA, Flynn JM, Moran ME, Phelps MA, Grever MR, Byrd JC. Activity of the cyclin-dependent kinase (CDK) inhibitor flavopiridol in relapsed, genetically high risk chronic lymphocytic leukemia (CLL). J Clin Oncol. 2008; 26:7007–7007. https://doi.org/10.1200/jco.2008.26.15_suppl.7007. [PubMed]
154. Flinn IW, Byrd JC, Bartlett N, Kipps T, Gribben J, Thomas D, Larson RA, Rai K, Petric R, Ramon-Suerez J, Gabrilove J, Grever MR. Flavopiridol administered as a 24-hour continuous infusion in chronic lymphocytic leukemia lacks clinical activity. Leuk Res. 2005; 29:1253–1257. https://doi.org/10.1016/j.leukres.2005.03.010. [PubMed]
155. Weide R. Bendamustine HCL for the treatment of relapsed indolent non-Hodgkin's lymphoma. Ther Clin Risk Manag. 2008; 4:727–732. https://doi.org/10.2147/TCRM.S3158. [PubMed]
156. Bigatti E, Canavesi A, Macdonald PL, Scarpitta F. Processes for preparing sunitinib and salts thereof. U.S. Patent US20090247767. 2009.
157. Friedberg JW, Sharman J, Sweetenham J, Johnston PB, Vose JM, LaCasce A, Schaefer-Cutillo J, De Vos S, Sinha R, Leonard JP, Cripe LD, Gregory SA, Sterba MP, et al. Inhibition of Syk with fostamatinib disodium has significant clinical activity in non-Hodgkin lymphoma and chronic lymphocytic leukemia. Blood. 2010; 115:2578–2585. https://doi.org/10.1182/blood-2009-08-236471. [PubMed]
158. Foss F, Advani R, Duvic M, Hymes KB, Intragumtornchai T, Lekhakula A, Shpilberg O, Lerner A, Belt RJ, Jacobsen ED, Laurent G, Ben-Yehuda D, Beylot-Barry M, et al. A Phase II trial of Belinostat (PXD101) in patients with relapsed or refractory peripheral or cutaneous T-cell lymphoma. Br J Haematol. 2015; 168:811–819. https://doi.org/10.1111/bjh.13222. [PubMed]
159. Yap TA, Yan L, Patnaik A, Fearen I, Olmos D, Papadopoulos K, Baird RD, Delgado L, Taylor A, Lupinacci L, Riisnaes R, Pope LL, Heaton SP, et al. First-in-man clinical trial of the oral pan-AKT inhibitor MK-2206 in patients with advanced solid tumors. J Clin Oncol. 2011; 29:4688–4695. https://doi.org/10.1200/JCO.2011.35.5263. [PubMed]
160. Ullah I, Wiley G. Enteric coated bead comprising ixabepilone, and preparation and administration there of. U.S. Patent US20060153917. 2006.
161. Pérez-Galán P, Dreyling M, Wiestner A. Mantle cell lymphoma: biology, pathogenesis, and the molecular basis of treatment in the genomic era. Blood. 2011; 117:26–38. https://doi.org/10.1182/blood-2010-04-189977. [PubMed]
162. Pession A, Masetti R, Kleinschmidt K, Martoni A. Use of clofarabine for acute childhood leukemia. Biologics. 2010; 4:111–118. https://doi.org/10.2147/btt.s10123.[PubMed]
163. Geissinger E, Bonzheim I, Roth S, Rosenwald A, Muller-Hermelink HK, Rudiger T. CD52 expression in peripheral T-cell lymphomas determined by combined immunophenotyping using tumor cell specific T-cell receptor antibodies. Leuk Lymphoma. 2009; 50:1010–1016. https://doi.org/10.1080/10428190902926981. [PubMed]
164. Manfredi MG, Ecsedy JA, Chakravarty A, Silverman L, Zhang M, Hoar KM, Stroud SG, Chen W, Shinde V, Huck JJ, Wysong DR, Janowick DA, Hyer ML, et al. Characterization of Alisertib (MLN8237), an investigational small-molecule inhibitor of aurora A kinase using novel *in vivo* pharmacodynamic assays. Clin Cancer Res. 2011; 17:7614–7624. https://doi.org/10.1158/1078-0432.CCR-11-1536. [PubMed]
165. Francisco JA, Cerveny CG, Meyer DL, Mixan BJ, Klussman K, Chace DF, Rejniak SX, Gordon KA, DeBlanc R, Toki BE, Law CL, Doronina SO, Siegall CB, et al. cAC10-vcMMAE, an anti-CD30-monomethyl auristatin E conjugate with potent and selective antitumor activity. Blood. 2003; 102:1458–1465. https://doi.org/10.1182/blood-2003-01-0039. [PubMed]
166. Revill P, Mealy N, Serradell N, Bolos J, Rosa E. Panobinostat. Drugs Future. 2007; 32:315. https://doi.org/10.1358/dof.2007.032.04.1094476.
167. Richon VM. Cancer biology: mechanism of antitumour action of vorinostat (suberoylanilide hydroxamic acid), a novel histone deacetylase inhibitor. Br J Cancer. 2006; 95:S2–S6. https://doi.org/10.1038/sj.bjc.6603463.
168. Genetech. Avastin-highlights of prescribing information. 2015.
169. Takahashi N, Watanabe Y, Maitani Y, Yamauchi T, Higashiyama K, Ohba T. p-Dodecylaminophenol derived from the synthetic retinoid, fenretinide: antitumor efficacy *in vitro* and *in vivo* against human prostate cancer and mechanism of action. Int J Cancer. 2008; 122:689–698. https://doi.org/10.1002/ijc.23154. [PubMed]
170. Robak T. GA-101, a third-generation, humanized and glyco-engineered anti-CD20 mAb for the treatment of B-cell lymphoid malignancies. Curr Opin Investig Drugs. 2009; 10:588–596. [PubMed]
171. Jurczak W, Kisiel E, Sawczuk-Chabin J, Centkowski P, Knopinska-Posluszny W, Khan O. The use of Yttrium-90 Ibritumomab Tiuxetan (90)Y-IT) as a consolidation therapy in high-risk patients with diffuse large B-cell lymphoma ineligible for autologous stem-cell transplantation. Contemp Oncol. 2015; 19:43–47. https://doi.org/10.5114/wo.2015.50012. [PubMed]
172. BC Cancer Agency. Carmustine. BC Cancer Agency Drug Manual. 2011.
173. Guidetti A, Carlo-Stella C, Locatelli SL, Malorni W, Pierdominici M, Barbati C, Mortarini R, Devizzi L, Matteucci P, Marchianò A, Lanocita R, Farina L, Dodero A, et al. Phase II study of sorafenib in patients with relapsed or refractory lymphoma. Br J Haematol. 2012; 158:108–119. https://doi.org/10.1111/j.1365-2141.2012.09139.x.
174. Fabricius HA, Stahn R. Serum-free and mitogen-free T-cell growth factor and process for making same. U.S. Patent US4464355. 1971.
175. Martin LA, Head JE, Pancholi S, Salter J, Quinn E, Detre S, Kaye S, Howes A, Dowsett M, Johnston SR. The farnesyltransferase inhibitor R115777 (tipifarnib) in combination with tamoxifen acts synergistically to inhibit MCF-7 breast cancer cell proliferation and cell cycle progression *in vitro* and *in vivo*. Mol Cancer Ther. 2007;

6:2458–2467. https://doi.org/10.1158/1535-7163.MCT-06-0452. [PubMed]

176. VanderMolen KM, McCulloch W, Pearce CJ, Oberlies NH. Romidepsin (Istodax, NSC 630176, FR901228, FK228, depsipeptide): a natural product recently approved for cutaneous T-cell lymphoma. J Antibiot. 2011; 64:525–531. https://doi.org/10.1038/ja.2011.35. [PubMed]

177. Ribas A. Tumor immunotherapy directed at PD-1. N Engl J Med. 2012; 366:2517–2519. https://doi.org/10.1056/NEJMe1205943. [PubMed]

178. Patnaik A, Appleman LJ, Mountz JM, Ramanathan RK, Beeram M, Tolcher AW, Papadopoulos KP, Lotze MT, Petro DP, Laymon C, Paige L, Rajagopalan P, Jeffers M, et al. A first-in-human phase I study of intravenous PI3K inhibitor BAY 80-6946 in patients with advanced solid tumors: Results of dose-escalation phase. J Clin Oncol. 2011; 29.

179. Haegebarth A, Haike K, Paul J, Mumberg D, Ziegelbauer K, Liu N. Potent *in vitro* and *in vivo* anti-tumor activity of PI3K inhibitor BAY 80-6946 and MEK inhibitor BAY 86-9766 in preclinical biliary tract cancer models. Cancer Res. 2012; 72:869. https://doi.org/10.1200/jco.2011.29.15_suppl.3035. [PubMed]

180. Grisafi D, Maestro A, Grumi C, Piazzoni L, Tirone G, Fiore W, Tessari R, Gianardi V, Gatti M, Tasca F, Generali D, Ravelli A, Lanza F, et al. Ibrutinib: from benchside to clinical implications. Med Oncol. 2015; 32:225. https://doi.org/10.1007/s12032-015-0669-9. [PubMed]

181. Zhang L, Pham LV, Newberry KJ, Ou Z, Liang R, Qian J, Sun L, Blonska M, You Y, Yang J, Lin X, Rollo A, Tamayo AT, et al. *In vitro* and *in vivo* therapeutic efficacy of carfilzomib in mantle cell lymphoma: targeting the immunoproteasome. Mol Cancer Ther. 2013; 12:2494–2504. https://doi.org/10.1158/1535-7163.MCT-13-0156. [PubMed]

182. De Ferra L, Zenoni M, Turchetta S, Anibaldi M, Ammirati E, Brandi P, Berardi G. Process for the synthesis of azacitidine and decitabine. U.S. Patent US20110245485. 2011.

183. Robert C, Ribas A, Wolchok JD, Hodi FS, Hamid O, Kefford R, Weber JS, Joshua AM, Hwu WJ, Gangadhar TC, Patnaik A, Dronca R, Zarour H, et al. Anti-programmed-death-receptor-1 treatment with pembrolizumab in ipilimumab-refractory advanced melanoma: a randomised dose-comparison cohort of a phase 1 trial. Lancet. 2014; 384:1109–1117. https://doi.org/10.1016/S0140-6736(14)60958-2. [PubMed]

184. Giles F, Rizzieri D, Karp J, Vey N, Ravandi F, Faderl S, Khan KD, Verhoef G, Wijermans P, Advani A, Roboz G, Kantarjian H, Bilgrami SF, et al. Cloretazine (VNP40101M), a novel sulfonylhydrazine alkylating agent, in patients age 60 years or older with previously untreated acute myeloid leukemia. J Clin Oncol. 2007; 25:25–31. https://doi.org/10.1200/JCO.2006.07.0961. [PubMed]

185. Gertz MA. Pomalidomide and myeloma meningitis. Leuk Lymphoma. 2013; 54:681–682. https://doi.org/10.3109/10428194.2012.723708. [PubMed]

186. Talpaz M, Shah NP, Kantarjian H, Donato N, Nicoll J, Paquette R, Cortes J, O'Brien S, Nicaise C, Bleickardt E, Blackwood-Chirchir MA, Iyer V, Chen TT, et al. Dasatinib in imatinib-resistant Philadelphia chromosome-positive leukemias. N Engl J Med. 2006; 354:2531–2541. https://doi.org/10.1056/NEJMoa055229. [PubMed]

187. Kavanagh JJ, Gershenson DM, Choi H, Lewis L, Patel K, Brown GL, Garcia A, Spriggs DR. Multi-institutional phase 2 study of TLK286 (TELCYTA, a glutathione S-transferase P1-1 activated glutathione analog prodrug) in patients with platinum and paclitaxel refractory or resistant ovarian cancer. Int J Gynecol Cancer. 2005; 15:593–600. https://doi.org/10.1111/j.1525-1438.2005.00114.x. [PubMed]

188. Etchin J, Sanda T, Mansour MR, Kentsis A, Montero J, Le BT, Christie AL, McCauley D, Rodig SJ, Kauffman M, Shacham S, Stone R, Letai A, et al. KPT-330 inhibitor of CRM1 (XPO1)-mediated nuclear export has selective anti-leukaemic activity in preclinical models of T-ALL and AML. Br J Haematol. 2013; 161:117–127. https://doi.org/10.1111/bjh.12231. [PubMed]

189. Zugmaier G, Klinger M, Schmidt M, Subklewe M. Clinical overview of anti-CD19 BiTE and *ex vivo* data from anti-CD33 BiTE as examples for retargeting T cells in hematologic malignancies. Mol Immunol. 2015; 67:58–66. https://doi.org/10.1016/j.molimm.2015.02.033. [PubMed]

190. Hutchinson CV, Dyer MJ. Breaking good: the inexorable rise of BTK inhibitors in the treatment of chronic lymphocytic leukaemia. Br J Haematol. 2014; 66:12–22. https://doi.org/10.1111/bjh.12895. [PubMed]

191. Reilly MP, Sinha U, André P, Taylor SM, Pak Y, DeGuzman FR, Nanda N, Pandey A, Stolla M, Bergmeier W, McKenzie SE. PRT-060318, a novel Syk inhibitor, prevents heparin-induced thrombocytopenia and thrombosis in a transgenic mouse model. Blood. 2011; 117:2241–2246. https://doi.org/10.1182/blood-2010-03-274969. [PubMed]

192. Evenou JP, Wagner J, Zenke G, Brinkmann V, Wagner K, Kovarik J, Welzenbach KA, Weitz-Schmidt G, Guntermann C, Towbin H, Cottens S, Kaminski S, Letschka T, et al. The Potent Protein Kinase C-Selective Inhibitor AEB071 (Sotrastaurin) Represents a New Class of Immunosuppressive Agents Affecting Early T-Cell Activation. J Pharmacol Exp Ther. 2009; 330:792–801. https://doi.org/10.1124/jpet.109.153205. [PubMed]

193. Incyte Corp. Jakafi prescribing information. 2016.

194. Padrnos L, Mesa RA. A closer look at pacritinib: a JAK2/FLT3 inhibitor for the treatment of myelofibrosis. Expert Opin Orphan Drugs. 2014; 2:725–733. https://doi.org/10.1517/21678707.2014.927761.
